# PQM-1 controls hypoxic survival via regulation of lipid metabolism

**DOI:** 10.1038/s41467-020-18369-w

**Published:** 2020-10-02

**Authors:** Thomas Heimbucher, Julian Hog, Piyush Gupta, Coleen T. Murphy

**Affiliations:** 1 Lewis-Sigler Institute for Integrative Genomics, Princeton University, Princeton, NJ 08544 USA; 2 Department of Molecular Biology, Princeton University, Princeton, NJ 08544 USA; 3 Bioinformatics and Molecular Genetics, Faculty of Biology, University of Freiburg, Freiburg, 79104 Baden-Wuerttemberg Germany

**Keywords:** Development, Gene expression, Molecular biology

## Abstract

Animals have evolved responses to low oxygen conditions to ensure their survival. Here, we have identified the *C. elegans* zinc finger transcription factor PQM-1 as a regulator of the hypoxic stress response. PQM-1 is required for the longevity of insulin signaling mutants, but surprisingly, loss of PQM-1 increases survival under hypoxic conditions. PQM-1 functions as a metabolic regulator by controlling oxygen consumption rates, suppressing hypoxic glycogen levels, and inhibiting the expression of the sorbitol dehydrogenase-1 SODH-1, a crucial sugar metabolism enzyme. PQM-1 promotes hypoxic fat metabolism by maintaining the expression of the stearoyl-CoA desaturase FAT-7, an oxygen consuming, rate-limiting enzyme in fatty acid biosynthesis. PQM-1 activity positively regulates fat transport to developing oocytes through vitellogenins under hypoxic conditions, thereby increasing survival rates of arrested progeny during hypoxia. Thus, while *pqm-1* mutants increase survival of mothers, ultimately this loss is detrimental to progeny survival. Our data support a model in which PQM-1 controls a trade-off between lipid metabolic activity in the mother and her progeny to promote the survival of the species under hypoxic conditions.

## Abstract (web summary)

Animals respond to hypoxic stress by adjusting metabolic processes to balance survival and reproduction. Here the authors identify the transcription factor PQM-1 as a metabolic regulator that balances hypoxic lipid and carbohydrate metabolism in *C. elegans* to limit somatic integrity and promote progeny survival.

## Introduction

A constant oxygen supply is essential to sustain the life of aerobic organisms, which have evolved multiple adaptive mechanisms to maintain the delicate balance between oxygen supply and demand. Impairment of this balance is associated with many age-related diseases, affecting pulmonary and cardiac function^1^ and increasing the economic burden of aging populations in modern societies. Consequently, understanding the defense mechanisms that animals have developed to protect against oxygen deprivation is important for the development of treatment strategies for human ischemia-related diseases and cancer.

*C. elegans* has been used as a model to study survival under a range of oxygen levels^2^ because it is well adapted to limited oxygen conditions; in fact, worms prefer 5–12% rather than atmospheric (21%) levels of oxygen^3,4^. In its natural habitat, *C. elegans* frequently encounters conditions where oxygen is depleted, because flooded soils can become hypoxic^5,6^. When exposed to very low oxygen levels (<0.3% O_2_), *C. elegans* enters a state of suspended animation^2^, a reversible hypometabolic state in which biological processes are largely slowed or even arrested. Intermediate levels of oxygen differentially affect transcriptional responses. The key hypoxic transcriptional regulator HIF-1 is required for development and survival of *C. elegans* at 0.5–1% oxygen (hypoxia), whereas it is not essential for survival in complete anoxia^7^. More recently, the zinc finger protein BLMP-1 was identified as a regulator of a HIF-1-independent hypoxic response based on a reporter screen performed with the hypoxia mimetic CoCl_2,_ which largely replicates the hypoxic state^8^.

Insulin/IGF-1 signaling (IIS) controls survival under various stresses, including hypoxia. Reduction-of-function mutations in the Insulin/IGF-1 receptor homolog *daf-2* protect *C. elegans* from high-temperature hypoxia and long-term anoxia^9,10^. The resistance against oxygen depletion is mediated by the FoxO transcription factor DAF-16, the major downstream transcriptional effector of IIS^11–15^.

We previously identified the C2H2-type zinc finger protein PQM-1 as an IIS-regulated DAF-16 antagonist and transcriptional regulator that is required for the exceptional longevity of *daf-2* mutants^15^. As reduction of IIS is highly protective for survival of nematodes exposed to hypoxic stress^9^, here we asked whether PQM-1, like DAF-2 and DAF-16, plays a role in hypoxic survival. Surprisingly, loss of *pqm-1*, unlike loss of *daf-16*, protects *C. elegans* from hypoxic stress. To understand the underlying mechanisms of this protection, we studied the transcriptional changes in *pqm-1* mutants under normoxia versus hypoxia, and discovered that alterations in carbohydrate and lipid metabolism are key to this survival. Ultimately, however, the loss of *pqm-1* under hypoxic stress is detrimental to future generations, suggesting that PQM-1 is an important component of multi-generational hypoxic survival through its regulation of key lipid metabolic genes.

## Results

### PQM-1 acts as a negative regulator of hypoxic survival independently of Insulin-like signaling

**Fig. 1:**
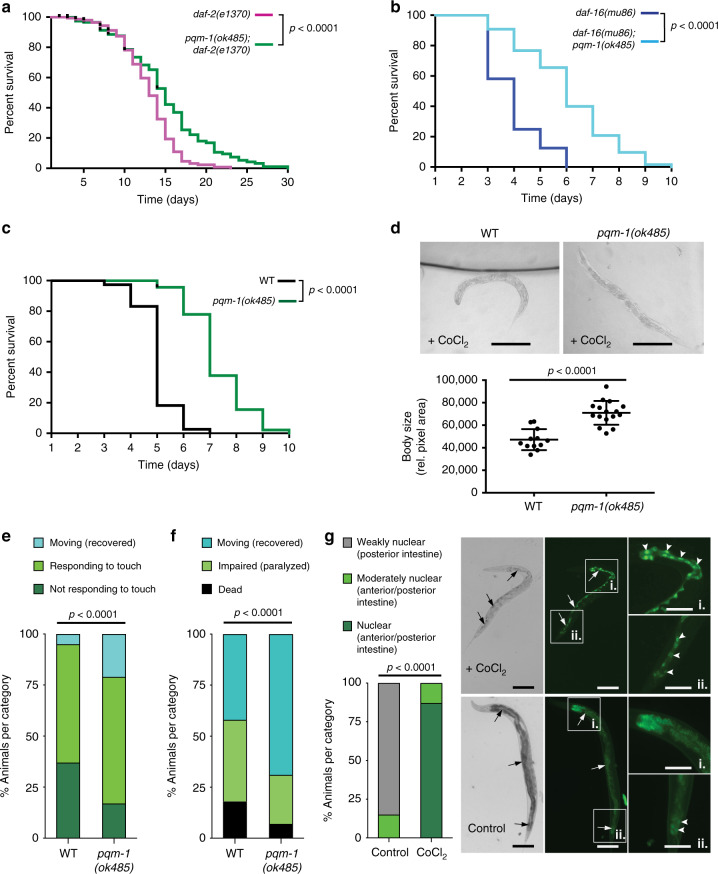
Loss of *pqm-1* increases survival and recovery under hypoxic conditions. **a**–**c** Survival analysis of animals exposed to 5 mM CoCl_2_ at the L4 larval stage. **a** *daf-2(e1370)* (*n* = 130), *pqm-1(ok485);daf-2(e1370)* (*n* = 99). **b** *daf-16(mu86)* (*n* = 51), *daf-16(mu86);pqm-1(ok485)* (*n* = 63). **c** WT (N2) (*n* = 70), *pqm-1(ok485)* (*n* = 45). Log-rank analysis (two-sided) (**a**–**c**). Three (**a**, **b**) and four (**c**) independent experiments were performed. **d** Shrinking of worms exposed to 5 mM CoCl_2_ at the L4 larval stage for 3 days and quantification of body size. Two-tailed *t*-test, mean ± SD, WT (N2) (*n* = 12), *pqm-1(ok485)* (*n* = 16); scale bars: 200 µm. Three independent experiments were performed. **e** Measurement of touch response of animals after exposure to <0.3% O_2_ in a hypoxic chamber for 16 h at 26 °C followed by an 8 h recovery period; WT (N2) (*n* = 156), *pqm-1(ok485)* (*n* = 196). **f** Survival analysis of animals after exposure to <0.3% O_2_ in a hypoxic chamber for 16 h at 26 °C followed by a one-day recovery period; WT (N2) (*n* = 137), *pqm-1(ok485)* (*n* = 162). **e**, **f** Chi-square analysis was performed, three independent experiments. **g** Nuclear localization analysis of a PQM-1::GFP fusion protein expressed in the intestine of worms exposed to 5 mM CoCl_2_ at the late L4 larval stage for 20 h until worms reach the adulthood stage. Arrows point to intestine; arrowheads point to intestinal nuclei in insets. Insets display anterior (i.) and posterior (ii.) intestinal regions. Representative images for the category “nuclear in anterior and posterior intestine” are displayed (CoCl_2_ condition, top). The category “weakly nuclear in posterior intestine” is represented by the control condition (bottom); scale bars: 100 µm, in insets: 50 µm. Chi-square analysis was performed; control condition (*n* = 54), CoCl_2_ condition (*n* = 30); three independent experiments.

We previously found that the zinc finger transcription factor PQM-1 is partially required for *daf-2* mutants’ extended longevity^15^, and thus we expected PQM-1 to also be required for *daf-2’s* extended survival in hypoxic conditions. Exposure of *C. elegans* to the hypoxia mimetic cobalt chloride (CoCl_2_) activates HIF-1 and mimics hypoxic responses^16^, allowing time course survival analysis under chemical hypoxia conditions^8^, whereas a typical hypoxic chamber experiment allows one survival time point after a normoxic recovery period. CoCl_2_ might mimic HIF dependent, chronic lower level of hypoxia in *C. elegans* and might have disease relevance for chronic lower level hypoxia in cancer cells. Counter to our expectations, *pqm-1(ok485);daf-2(e1370)* double mutants exposed to 5 mM CoCl_2_ survived significantly longer than did *daf-2(e1370)* worms (Fig. 1a). (All animals were synchronized at the L4 larval stage for survival analyses, as we previously found that *pqm-1* mutants develop more slowly than do wild-type animals^15^.) Together, these results suggest that PQM-1 activity may limit the resistance of *daf-2(e1370)* animals exposed to hypoxic stress.

The transcriptional outputs of IIS are largely mediated by the FOXO transcription factor DAF-16^14,15^. Superficially, *daf-16* and *pqm-1* act similarly in longevity regulation, as the mutants both have normal/short lifespans and both transcription factors are required for *daf-2’s* longevity. However, under CoCl_2_-mediated hypoxia, loss of *pqm-1* greatly increased the survival of a *daf-16(mu86)* null mutant (Fig. 1b and Supplementary Fig. 1), suggesting that PQM-1 acts independently of DAF-16 to regulate hypoxic survival. *pqm-1(ok485)* loss-of-function mutants survived longer than wild-type animals (Fig. 1c and Supplementary Fig. 1) and maintained a largely normal morphology, whereas wild-type worms shrank after 3 days of exposure to the hypoxia mimetic (Fig. 1d). Our data indicate that PQM-1 acts as a negative regulator of somatic integrity and survival under CoCl_2_-induced chemical hypoxia stress.

To verify our findings from CoCl_2_-mediated hypoxia to true hypoxia, we tested the recovery rate of wild-type and *pqm-1(ok485)* mutants after treatment in a hypoxic chamber. When animals were exposed to <0.3% O_2_ for 16 h at 26 °C^17^ followed by an 8 h normoxic recovery period, *pqm-1(ok485)* mutants exited suspended animation earlier than did wild-type animals (Fig. 1e). Following a normoxic one-day recovery period to identify dead worms, *pqm-1(ok485)* mutants survived and recovered from oxygen depletion better than did wild-type animals (Fig. 1f), indicating that loss of PQM-1 function improved survival under true hypoxia.

To address whether hypoxic stress can activate PQM-1 as transcription factor, we exposed worms expressing a PQM-1::GFP fusion protein to 5 mM CoCl_2_ and found that hypoxic stress promoted the nuclear localization of PQM-1::GFP in the intestine of worms (Fig. 1g). Together, these results suggest that hypoxic stress appears to activate PQM-1 by enhancing its nuclear localization to confer its function as a transcriptional regulator in the hypoxic stress response.

### PQM-1 is a transcriptional regulator of the hypoxic stress response

**Fig. 2:**
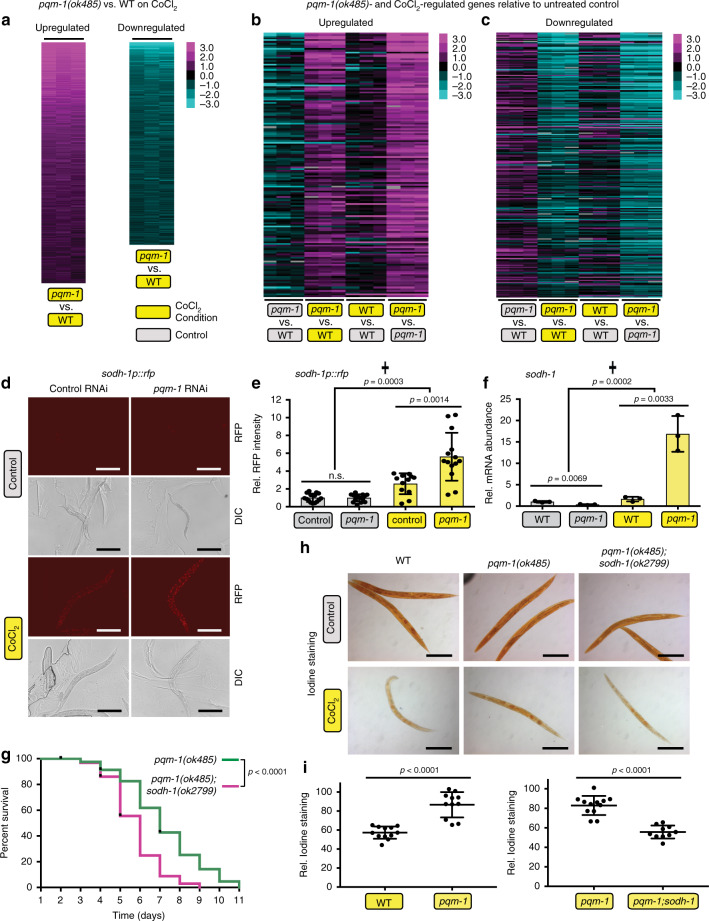
Glycogen metabolism is altered under hypoxia via PQM-1’s regulation of *sodh-1*. **a** One-class SAM analysis of gene expression changes in *pqm-1(ok485)* mutants +CoCl_2_ versus wild type (WT) +CoCl_2_ identified 1650 upregulated and 629 downregulated genes (Supplementary Data 2). **b**, **c** *pqm-1-* and CoCl_2_-dependent upregulated (**b**) and downregulated genes (**c**). Two-class SAM of *pqm-1(ok485)* +CoCl_2_ versus WT +CoCl_2_ compared to untreated *pqm-1(ok485)* versus untreated WT identified 152 upregulated and 243 downregulated genes (Supplementary Data 3). Heat maps depict individual genes (rows) ranked by SAM score (median FDR < 0.01), with columns representing individual microarrays (**a**–**c**). Animals were exposed to 5 mM CoCl_2_ for 6 h at early adulthood (**a**–**c**). **d** *sodh-1p::rfp* reporter activity after 20 h of 5 mM CoCl_2_ exposure at a late larval (L4) stage; **e** quantification of reporter activity in (**d**); empty-vector control (*n* = 15) and *pqm-1(ok485)* (*n* = 16) without CoCl_2_ (gray bars); empty-vector control (*n* = 12) and *pqm-1(ok485)* (*n* = 14) +CoCl_2_ (yellow bars); three independent experiments. **f** *pqm-1-* and CoCl_2_-dependent expression changes of endogenous *sodh-1* determined by RT-qPCR (*n* = 3 biological replicates) following 5 mM CoCl_2_ exposure for 6 h at early adulthood. Two-tailed *t*-test and Two-way ANOVA (✝) (**e**, **f**), mean ± SD, ns = not significant. **g** Survival analysis of *pqm-1(ok485)* (*n* = 67) and *pqm-1(ok485);sodh-1(ok2799)* mutants (*n* = 72) exposed to 5 mM CoCl_2_ at the L4 larval stage. Log-rank analysis (two-sided). Three independent experiments were performed. **h**, **i** Glycogen levels of worms  subjected to 5 mM CoCl_2_ followed by iodine vapor staining (**h**); **i** quantification of glycogen levels in (**h**); wild type (*n* = 12), *pqm-1(ok485)* (*n* = 11 [left in (**i**)], *n* = 12 [right in (**i**)]) and *pqm-1(ok485);sodh-1(ok2799)* mutants (*n* = 10). Scale bars: 200 µm (**d**, **h**). Two-tailed *t*-test, mean ± SD; three independent experiments.

Under normoxia, the transcription factor PQM-1 controls gene expression programs that regulate development, growth, and reproduction^15^. To identify genes altered in chemical hypoxia, we carried out three types of analyses: first, we performed an analysis of genes that change their expression in wild-type animals treated with CoCl_2_ (Supplementary Fig. 2a and Supplementary Data 1; one-class SAM, 366 upregulated and 56 downregulated genes). Second, we directly compared *pqm-1* and wild-type animals that had both been exposed to CoCl_2_ to identify those genes that provide *pqm-1* a survival advantage under hypoxic conditions (Fig. 2a and Supplementary Data 2; one-class SAM, 1650 upregulated and 629 downregulated genes). Third, we carried out a two-class SAM analysis in comparisons of wild-type and *pqm-1* worms both treated with CoCl_2_ vs untreated to further identify potential *pqm-1* targets that increase survival under hypoxic conditions (Fig. 2b–c and Supplementary Data 3). We found that genes associated with response to heat shock (*hsp-16*, *hsp-70*), metal (*numr* and *cdr*), and infection (*irg-1*, *-2*), as well as glutathione S-transferases (*gst*), several DAF-16 target genes (*dod-22, -17, -24*), and PQM-1/DAF-16 regulated targets (C32H11 and F55G11)^14^ are upregulated, indicating that the animals are responding to stress conditions. Several cytochrome P450s (*cyp-35A2, -25A1, -14A2, -25A2, -34A9*), short-chain dehydrogenases (*dhs-25, -20, -2*) and lipid metabolism genes (e.g., *lips-14, elo-6, acdh-2, elo-5*) are downregulated under chemical hypoxia conditions (Supplementary Fig. 2a and Supplementary Data 1; FDR < 0.01), indicating metabolic changes. Gene Ontology analysis (Supplementary Fig. 2b) suggests that worms subjected to CoCl_2_ hypoxic conditions affect immune and defense response, ER signaling, aging and lifespan regulation, and lipid metabolism. *pqm-1* and CoCl_2_-dependent upregulated gene categories are enriched for immune, defense, and stress responses, while downregulated genes are associated with the response to xenobiotic stimuli, oxidation-reduction processes, lipid metabolism, and transmembrane transport (Supplementary Fig. 2c, d and Supplementary Data 2). GO analysis of the *pqm-1* and CoCl_2_-dependent upregulated gene sets, which were analyzed for expression changes relative to wild-type animals and untreated controls using two-class SAM, revealed an enrichment for oxidation-reduction processes and the innate immune response (Supplementary Fig. 3a and Supplementary Data 3). The GO term “metabolism” is highly significant in the downregulated gene set (Supplementary Fig. 3b and Supplementary Data 3), with fatty acid metabolism, flavonoid-related processes, and transmembrane transport GO terms statistically enriched.

Carbohydrate metabolism is critical for *C. elegans* survival under conditions where oxygen is limited^10,18–20^. *sodh-1/dod-11* was one of the top two-class SAM hits for upregulated genes in a *pqm-1(ok485)* mutant challenged with CoCl_2_ (Supplementary Fig. 3c and Supplementary Data 3). Sorbitol dehydrogenase (SODH) enzymatic activity converts sorbitol, the sugar alcohol form of glucose, into fructose^21^. *sodh-1/dod-11* was previously identified as a downstream target of the IIS/DAF-16 pathway and is required for the long lifespan of *daf-2(lf*) mutants^14^; moreover, it is a key enzyme for starvation-induced aggregation of *C. elegans* and ethanol metabolism^22^ and is upregulated when worms are exposed to transient hypoxia^23^. The promotor region of *sodh-1/dod-11* contains two PQM-1 binding sites (DAEs) located within 1000 bp upstream of the translational start site, suggesting that it functions as a direct PQM-1 target gene^14,24^.

We confirmed the upregulation of *sodh-1/dod-11* observed in our two-class SAM using a fluorescent reporter: *sodh-1p::rfp* is induced when *pqm-1* was reduced by RNAi-mediated knock down in worms treated with CoCl_2_ (Fig. 2d, e). In addition, *sodh-1* endogenous transcript levels were upregulated in a *pqm-1(ok485)* mutant exposed to chemical hypoxia (Fig. 2f), suggesting that PQM-1 likely normally represses *sodh-1* expression. Reduction of *sodh-1* in *pqm-1(ok485)* mutants significantly reduced CoCl_2_ survival (Fig. 2g), indicating that *sodh-1* is necessary for *pqm-1’s* enhanced hypoxic survival.

The upstream regulatory region of *sodh-1/dod-11* is enriched for DAF-16 binding sites (DBEs) in addition to the two PQM-1 binding sites (DAEs)^14,24^. DAF-16 is required for *sodh-1* mediated transcriptional activation in *daf-2(lf)* mutants based on the *sodh-1p::rfp* fluorescent reporter^24^. Counter our expectations, however, we found that loss of *daf-16* increased the expression of the *sodh-1p::rfp* reporter in wild-type worms when IIS functions at normal physiological levels and in chemical hypoxia (Supplementary Fig. 3f–h). These data indicate that DAF-16 could act as a repressor along with PQM-1 to downregulate *sodh-1* expression in regular growth conditions or hypoxic stress.

Previous studies have demonstrated that sorbitol, the substrate of SODH-1, can be metabolized to glycogen in diapause eggs of insects (*Bombyx mori*) depending on the activity of SODH-1^25,26^. The ability to store carbohydrates such as glycogen correlates with increased survival of *C. elegans* in oxygen-deprived conditions^18,20^. To test the role of *pqm-1* and its downstream regulated target *sodh-1* in hypoxic carbohydrate metabolism, we measured glycogen levels. *pqm-1(ok485)* mutants maintain glycogen at higher levels than wild-type animals challenged with CoCl_2_, while *pqm-1(ok485);sodh-1(ok2799)* double mutants had reduced glycogen levels (Fig. 2h, i and Supplementary Fig. 3d, e), suggesting that *pqm-1’s* increased hypoxia survival is linked to its SODH-1-dependent increased glycogen levels.

### PQM-1 activity regulates lipid levels in hypoxia

**Fig. 3:**
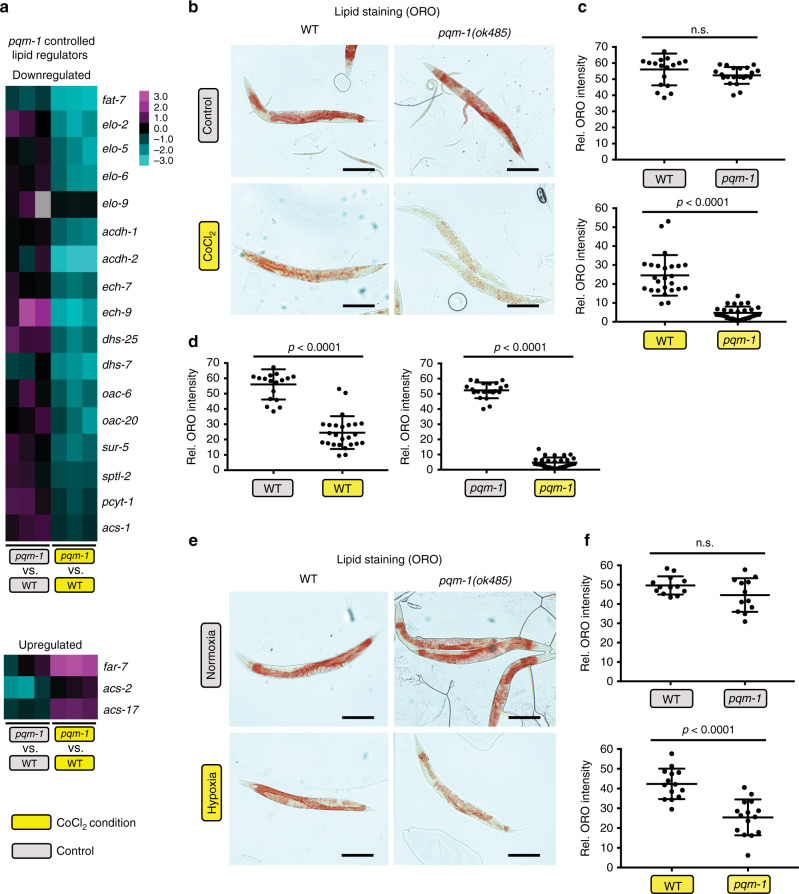
PQM-1 controls expression of lipid regulators and positively regulates hypoxic fat levels. **a** Expression based analysis of *pqm-1*-dependent regulators implicated in hypoxic lipid metabolism. Two-class SAM (*pqm-1(ok485)* +CoCl_2_ versus WT +CoCl_2_ compared to *pqm-1(ok485)* −CoCl_2_ versus WT −CoCl_2_) identified downregulated (top) and upregulated (bottom) lipid regulators. **b**–**f** Oil Red O-based lipid staining of animals subjected to control conditions, chemical hypoxia (2.5 mM CoCl_2_, **b**–**d**), and real hypoxia (0.4% oxygen, **e**, **f**) at the early day-1 of adulthood stage for 44 h followed by Oil Red O-based lipid staining (**b**, **e**) and whole worm quantification of fat levels (**c**, **d**, **f**). Animals were synchronized at the L4 larval stage for adult analyses. Two-tailed *t*-test, mean ± SD, **c**, **d**) WT (N2) (*n* = 18) and *pqm-1(ok485)* (*n* = 21) in control condition; WT (N2) (*n* = 25) and *pqm-1(ok485)* (*n* = 29) in CoCl_2_ condition. **f** WT (N2) (*n* = 13) and *pqm-1(ok485)* (*n* = 12) in control condition; WT (N2) (*n* = 14) and *pqm-1(ok485)* (*n* = 15) in hypoxic condition; ns = not significant; scale bars: 200 µm (**b**, **e**). Four (**b**–**d**) and two (**e**, **f**) independent experiments were performed.

The CoCl_2_-induced *pqm-1*-differentially-expressed gene list is enriched for lipid metabolism regulators. The downregulated genes included fatty acid elongases (*elo-2, -5, -6*, and *-9*), the stearoyl-CoA fatty acid desaturase *fat-7*, and enzymes predicted to function in fatty acid β-oxidation (*acdh-1*, *acdh-2, ech-7* and *-9*) (Fig. 3a, top), while upregulated genes included the fatty acid and retinoid-binding protein *far-7* and the acyl-CoA synthetases *asc-2* and *asc-17* (Fig. 3a, bottom). Oil Red O (ORO) staining, which detects neutral triglycerides and lipids, revealed no significant differences between wild type and *pqm-1* mutants under control conditions (Fig. 3b–c, top), but CoCl_2_ treatment for 44 h induced fat loss relative to untreated controls (Fig. 3b, d, left); this lipid loss was significantly more pronounced in *pqm-1(ok485)* mutants challenged with CoCl_2_ (Fig. 3b, d, right), suggesting that PQM-1 normally acts as a positive regulator of lipid level maintenance. Similarly, *pqm-1* mutants exposed to 0.4% O_2_ (hypoxia) for 44 h exhibited a significant reduction in fat levels compared to hypoxic wild-type animals (Fig. 3e, f), confirming that CoCl_2_ conditions mimic true hypoxia for fat metabolism. An additional, PQM-1-independent mechanism might be implicated in maintaining hypoxic fat mass, as wild-type animals also lose fat when challenged with the hypoxia mimetic. In some individual hypoxic *pqm-1(ok485)* animals, lipid levels were strongly depleted in the intestinal tissue (Fig. 3e), and fat was only detected in the eggs located in the uterus of *pqm-1(ok485)* mutants. These results suggest that PQM-1 acts as an essential intestinal metabolic regulator, promoting lipid levels under oxygen depletion.

### FAT-7 functions as a downstream PQM-1 target in hypoxic lipid metabolism

**Fig. 4:**
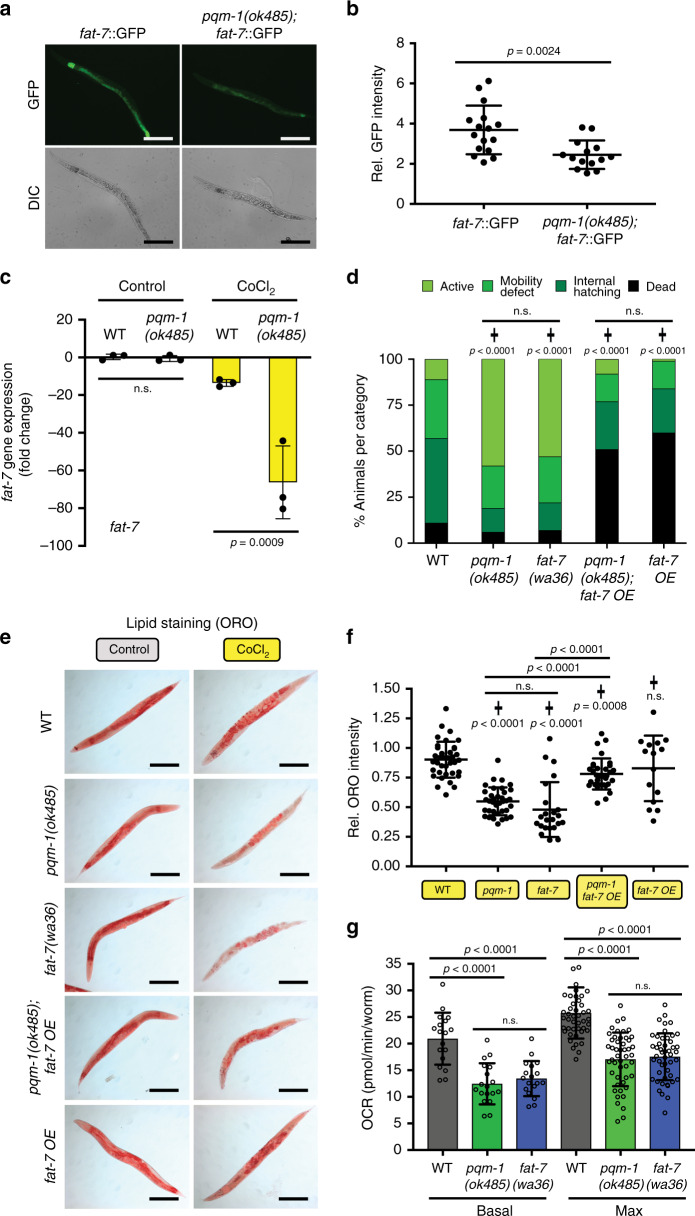
FAT-7 functions as a downstream PQM-1 target regulating hypoxic fat levels, oxygen consumption rates, and survival. **a**, **b** PQM-1 positively regulates *fat-7* expression in hypoxic conditions based on a *fat-7p*::*fat-7*::GFP translational reporter (**a**) and its quantification (**b**); *fat-7*::GFP (*n* = 16), *pqm-1(ok485);fat-7*::GFP (*n* = 14). Animals were exposed to 5 mM CoCl_2_ at a late larval (L4) stage for 24 h. Three independent experiments. **c** *pqm-1-* and CoCl_2_-dependent regulation of endogenous *fat-7* determined by RT-qPCR (*n* = 3 biological replicates) following 5 mM CoCl_2_ exposure for 20 h at early adulthood. Two-tailed *t*-test (**b**, **c**), mean ± SD, ns = not significant. **d** Survival and matricide analysis of indicated *C. elegans* strains exposed to 5 mM CoCl_2_ at the early adulthood stage (early day-1 of adulthood stage) for 50 h. (✝) indicates mutant versus wild type strain, Chi-square analysis; WT (N2) (*n* = 136), *pqm-1(ok485)* (*n* = 87), *fat-7(wa36)* (*n* = 76)*, pqm-1(ok485);fat-7* OE (*n* = 61), *fat-7* OE (*n* = 62); three independent experiments. **e** Oil Red O-based lipid staining of animals subjected to control and chemical hypoxia conditions. Animals were exposed to 2.5 mM CoCl_2_ at the early day-1 of adulthood stage for 44 h followed by Oil Red O staining (**e**) and whole worm quantification of fat levels (**f**). Quantification values were normalized to the mean value of WT control without CoCl_2_ (Supplementary Fig. 4e). WT (*n* = 37), *pqm-1(ok485)* (*n* = 36), *fat-7(wa36)* (*n* = 23)*, pqm-1(ok485);fat-7* OE (*n* = 32), *fat-7* OE (*n* = 15); scale bars: 200 µm (**a**, **e**); three independent experiments. **g** Measurement of oxygen consumption rates. The uncoupler FCCP was injected twice (10 µM each injection) to obtain maximal oxygen consumption rates. For each strain six biological repeats, each consisting of approximately 20–25 worms, have been repeatedly measured to obtain OCR values (source data file). Three independent experiments were performed. Two-tailed *t*-test (**f**, **g**), mean ± SD, (✝) indicates mutant versus wild type strain (**f**); ns = not significant. Animals were synchronized at the L4 larval stage for adult analyses.

The stearoyl-CoA desaturase *fat-*7 was the most significantly downregulated lipid regulator upon exposure to chemical hypoxia (Supplementary Fig. 4a and Supplementary Data 4). This suggests that *fat-7* is an essential transcriptional target of PQM-1 and that reduction of *fat-7* might be important for *pqm-1(ok485)*-mediated hypoxic fat loss and subsequent survival. FAT-7 is a stearoyl-CoA desaturase that catalyzes a critical rate-limiting step in fatty acid biosynthesis, desaturating stearate [CH_3_(CH2)_16_COO^−^] to mono-unsaturated oleate [CH_3_(CH_2_)_7_CH=CH(CH_2_)_7_COO^−^], which requires oxygen as an electron acceptor (Supplementary Fig. 4b). To verify our transcriptional results, we examined the expression of a *fat-7p::fat-7::gfp* translational reporter^27^ in *pqm-1* mutants, and found that fluorescence is significantly reduced in chemical hypoxia (Fig. 4a, b). Quantification of endogenous *fat-7* transcript levels revealed a downregulation of *fat-7* in *pqm-1* mutants after 6 h of CoCl_2_ exposure (Supplementary Fig. 4c). The reduction of *fat-7* transcript level was more pronounced after 20 h of chemical hypoxia (Fig. 4c), although already wild-type worms exposed to CoCl_2_ displayed a slight *fat-7* downregulation under these conditions, indicating that other regulators in addition to PQM-1 control *fat-7* expression in chemical hypoxia. Together, these data point to an important role of PQM-1 in *fat-7* transcriptional regulation in hypoxic conditions.

We tested the survival of the *fat-7(wa36)* loss-of-function mutant under CoCl_2_ conditions, and found that *fat-7* mutants displayed a moderate increase in survival when exposed to CoCl_2_ at a late larval (L4) stage (Supplementary Fig. 4d). When challenged with CoCl_2_ at the early adulthood stage, which promotes matricide in wild-type worms, *fat-7* loss-of-function mutants largely phenocopied the beneficial effect of *pqm-1* ablation in hypoxic survival (Fig. 4d). After 50 h of CoCl_2_ treatment, approximately half of *fat-7* mutants were still actively crawling on plates, which was similar to the mobility of *pqm-1(ok485)* animals under chemical hypoxia, and strikingly different from the immobility that wild-type animals display under these conditions (Fig. 4d). By contrast, reintroducing *fat-7* activity into a *pqm-1* loss-of-function mutant by overexpressing a *fat-7p::fat-7::gfp* translational reporter (“*fat-7 OE*”) completely reversed the beneficial effects of a *pqm-1(ok485)* mutant on hypoxic mobility and survival (Fig. 4d). Overexpression of FAT-7 in wild-type and *pqm-1* CoCl_2_-treated worms also caused high rates of matricide and death.

Next, we asked what role FAT-7 plays in lipid regulation under hypoxic conditions. *fat-7(wa36)* mutants under normoxia did not display decreased fat levels relative to wild-type animals (Fig. 4e and Supplementary Fig. 4e), but lack of *fat-7* activity in chemical hypoxia caused a reduction of lipid levels, similar to those observed in *pqm-1(ok485)* mutants (Fig. 4e, f). To determine whether the lipid-loss phenotype of *pqm-1* mutants is primarily caused by a downregulation of fatty acid synthesis through decreased *fat-7* expression, we performed a rescue experiment by re-expressing *fat-7* under its own promoter (*fat-7p::fat-7::gfp* translational reporter) and measuring lipid levels. Expression of *fat-7* largely restored lipid levels of *pqm-1* mutants exposed to the hypoxia mimetic (Fig. 4e, f), but did not significantly change lipid levels in *pqm-1* mutants relative to wild type animals under control conditions (Fig. 4e and Supplementary Fig. 4e). Thus, *pqm-1* partially controls hypoxic lipid levels through *fat-7*-mediated lipid biosynthesis.

As PQM-1 was initially identified as a transcriptional regulator downstream of IIS^15^, we analyzed whether PQM-1 controls hypoxic fat levels in the Insulin/IGF-1 receptor mutant *daf-2(e1370)*. Loss of *pqm-1* did not reduce fat levels of *daf-2(e1370)* mutants exposed to CoCl_2_ as it was observed for *pqm-1* mutants versus wild-type worms (Supplementary Fig. 4g). Both *daf-2(e1370)* and *pqm-1(ok485);daf-2(e1370)* mutants displayed increased amount of lipids relative to wild-type animals in control conditions and chemical hypoxia (Supplementary Fig. 4f, g). *pqm-1(ok485);daf-2(e1370)* mutants survive longer than *daf-2(e1370)* animals in hypoxic stress (Fig. 1a), but do not seem to reduce hypoxic lipid levels as *pqm-1(ok485)* versus wild-type worms. Thus, it is likely that processes in addition to fat metabolism might contribute to *pqm-1* loss-of-function mediated survival extension when IIS is compromised.

Next, we tested whether IIS regulates *fat-7* expression, and found that *fat-7p::fat-7::gfp* levels were suppressed in a *daf-2(e1370)* mutant compared to wild-type worms under control conditions (Supplementary Fig. 4h, i). Loss of *daf-16* function resulted in an increase of the *fat-7p::fat-7::gfp* reporter signal in *daf-2* mutants in both control and CoCl_2_ conditions (Supplementary Fig. 4h–j), indicating that DAF-16 activity (either directly or indirectly via PQM-1) represses *fat-7* when IIS is downregulated. A similar reporter de-repression was detected in wild-type worms on *daf-16* RNAi and CoCl_2_ versus wild-type control worms exposed to CoCl_2_. Loss of *pqm-1* in a *daf-2(e1370)* mutant increased the *fat-7p::fat-7::gfp* reporter activity (Supplementary Fig. 4h–j), indicating that *pqm-1* may mediate repression of *fat-7* expression when IIS is downregulated. In summary, our data suggest a *fat-7*-independent mechanism might promote elevated lipid levels in *daf-2* mutants, as DAF-16 represses *fat-7* transcription when IIS is reduced. An additional delta-9 desaturase, the palmitoyl-CoA desaturase *fat-5*, is involved in lipid biosynthesis of *daf-2* mutants^28^, which may provide an alternative mechanism for the maintenance of fat levels when IIS is compromised.

### *fat-7* mutants mimic *pqm-1* mutant reduction of oxygen consumption rates

We hypothesized that animals that survive in low oxygen conditions might do so by reducing their oxygen metabolism. We measured the basal and maximum oxygen consumption rates (OCR) of wild-type and *pqm-1* worms, and found that loss of *pqm-1* diminished both the basal and the maximal OCR (Fig. 4g). We then found that *fat-7(wa36)* loss-of-function mutants reduced oxygen consumption to levels comparable to *pqm-1(ok485)* mutants (Fig. 4g). Re-expressing *fat-7* in a *pqm-1(ok485)* mutant partially restored the basal OCR (Supplementary Fig. 4k). These data indicate that changes in *fat-7* expression affect oxygen consumption in worms. Together, our data suggest that *pqm-1* mutants metabolize oxygen at lower rates due to decreased FAT-7 levels, which, in turn, may decrease the negative impact of low oxygen conditions on the worms. *fat-7* appears to be an important target downstream of PQM-1 in the hypoxic response and in *pqm-1’s* oxygen utilization.

### PQM-1 promotes progeny formation and survival in hypoxic stress

Our findings suggested that normal PQM-1 activity is detrimental for hermaphrodites exposed to hypoxic stress through its promotion of fat metabolism and subsequent reduction of survival. Previously, trade-offs between somatic survival and progeny production have been linked to reallocation of lipid stores from the soma to the germline during nutrient and oxidative stress^29^. Therefore, we investigated PQM-1’s effect on progeny survival and their supply of lipids under hypoxic conditions.

**Fig. 5:**
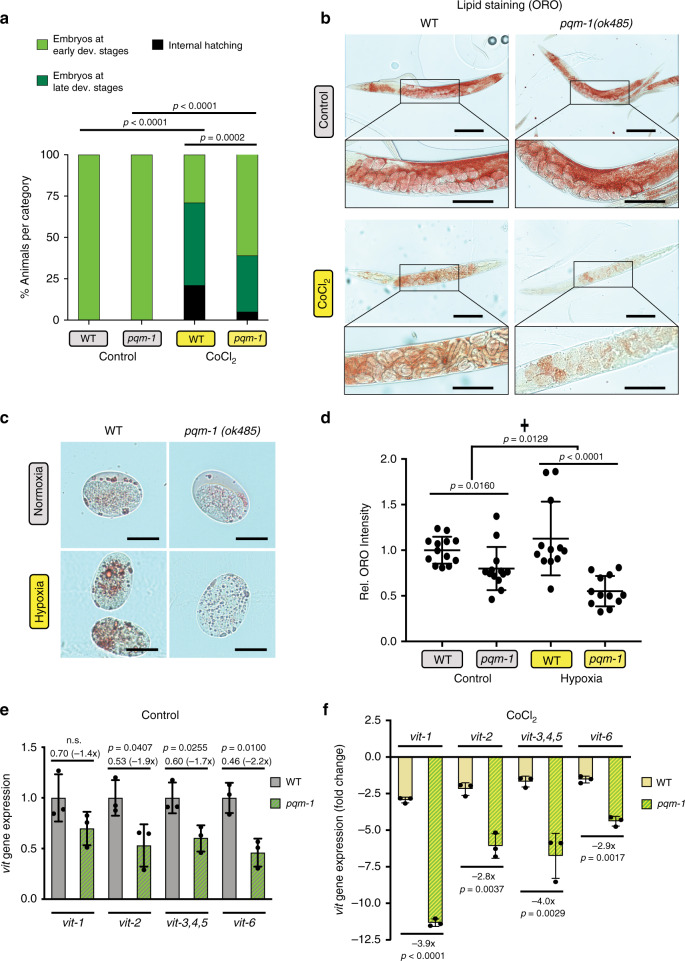
PQM-1 activity regulates vitellogenin expression and lipid content of progeny in hypoxic stress. **a** Quantification of matricide and embryonal stages in the uterus of hermaphrodites from ORO stained animals in (**b**). WT (N2) (*n* = 69) and *pqm-1(ok485)* (*n* = 77) in control condition; WT (N2) (*n* = 70) and *pqm-1(ok485)* (*n* = 65) in CoCl_2_ condition; Chi-square analysis was performed; three independent experiments. **b** Oil Red O-based lipid staining displaying progeny in parental hermaphrodites. Parental hermaphrodites were Oil Red O stained at the day-1 of adulthood stage (control). CoCl_2_ challenged hermaphrodites were exposed to 2.5 mM CoCl_2_ at the day-1 of adulthood stage for 44 h followed by Oil Red O staining. Animals were synchronized at the L4 larval stage for adult analyses. Scale bars: 200 µm, in inset: 100 µm. **c**, **d** Lipid content of eggs (**c**) dissected from Oil Red O stained hermaphrodites challenged with 0.4% oxygen for 44 h and quantification of fat levels in eggs (**d**). WT (N2) eggs (*n* = 13) and *pqm-1(ok485)* eggs (*n* = 13) in control condition; WT (N2) eggs (*n* = 12) and *pqm-1(ok485)* eggs (*n* = 12) in hypoxic condition; scale bars: 25 µm. Two-tailed *t*-test, Two-way ANOVA (✝), mean ± SD, three independent experiments. **e**, **f** *pqm-1-* and CoCl_2_-dependent regulation of endogenous vitellogenins determined by RT-qPCR (*n* = 3 biological replicates) for control condition (**e**) and condition of 5 mM CoCl_2_ exposure (**f**). Reduction of *vit* gene expression in *pqm-1* mutants relative to WT is indicated below the *p*-value and the corresponding fold change value is displayed in brackets (**e**). Quantification values were normalized to mean value of WT control without CoCl_2_ (**e**, **f**). Animals were exposed to 5 mM CoCl_2_ for 6 h at early adulthood. Two-tailed *t*-test (**e**, **f**), mean ± SD, ns = not significant.

To study the effect of hypoxia on progeny development, we examined the formation of embryos in the uterus of hermaphrodites exposed to CoCl_2_. Usually *C. elegans* lays eggs that have developed to the gastrula stage, but chemical hypoxia caused an egg-laying defect, in utero retention of embryos that had developed further than normoxic control embryos (Fig. 5a, b). Moreover, hypoxic wild-type animals contained substantial amounts of lipid that appeared to reallocate to the progeny (Fig. 5b). By contrast, hypoxic *pqm-1* mutants contained less fat, embryos in the uterus were found largely at earlier developmental stages, and the mutants displayed less internal hatching compared to wild-type hypoxic hermaphrodites (Fig. 5a, b).

To examine the lipid levels in embryos, we quantified ORO staining of eggs in hermaphrodites challenged with the real hypoxia and the hypoxia mimetic (Fig. 5c, d and Supplementary Fig. 5a, b). Eggs dissected out of *pqm-1* mutants contained significantly less fat than embryos of wild-type animals, both in control conditions and when exposed to CoCl_2_ (Supplementary Fig. 5a, b). When eggs were dissected out of ORO-stained hermaphrodites challenged with hypoxia, the lipid content of embryos originating from *pqm-1* mutants was diminished relative to hypoxia-treated wild-type embryos (Fig. 5c, d). Our data suggest that PQM-1 activity enhances lipid accumulation in embryos in both control and hypoxic environments.

Lipids are transported from the *C. elegans* intestine to its developing oocytes by vitellogenins, which are precursor proteins of egg yolk^30^. Vitellogenins are taken up by oocytes through receptor-mediated endocytosis^31^, and are present in developing embryos to promote their survival under stress conditions such as starvation^32,33^. Our microarray data revealed a transcriptional downregulation of vitellogenins in *pqm-1* mutants exposed to CoCl_2_ (Supplementary Fig. 5c, condition 4 in heatmap, Supplementary Data 2). A *pqm-1-* and CoCl_2_-dependent regulation of vitellogenins was further validated by quantifying endogenous vitellogenin transcript levels. *pqm-1* loss moderately downregulated vitellogenin expression relative to wild type in control condition (Fig. 5e). Vitellogenin expression was already slightly decreased in wild-type animals treated with CoCl_2_ for 6 h (Fig. 5f), however, a downregulation was more pronounced in CoCl_2_-exposed *pqm-1* mutants for certain vitellogenins such as *vit-1* and *vit-3,4,5*.

Our data point to an important role of PQM-1 in promoting vitellogenin expression in hypoxic stress environments, but the data also suggest that additional vitellogenin regulators could contribute. The transcription factors UNC-62 and CEH-60 co-regulate vitellogenin expression through binding to the promotor regions of vitellogenins^34^. To test whether these transcription factors additively regulate vitellogenin gene expression, we treated wild type and *pqm-1* mutants with either *unc-62* or *ceh-60* RNAi. *vit* gene expression in *pqm-1* mutants was not affected by reduction of either TF in control conditions (Supplementary Fig. 5d, f), or by loss of *unc-62* in CoCl_2_ exposure (Supplementary Fig. 5e), but reduction of *ceh-60* in *pqm-1* mutants slightly abrogated this change in *vit* expression under CoCl_2_ conditions (Supplementary Fig. 5g). ChIP-Seq data indicate that PQM-1 binds to upstream regulatory regions of *ceh-60*^34^. Although *ceh-60* transcript levels were upregulated in chemical hypoxia, loss of *pqm-1* function in hypoxia did not compromise *ceh-60* expression relative to wild-type hypoxic animals (Supplementary Fig. 6a). However, *unc-62* transcript levels decreased in *pqm-1* mutants under hypoxic stress (Supplementary Fig. 6b).

**Fig. 6:**
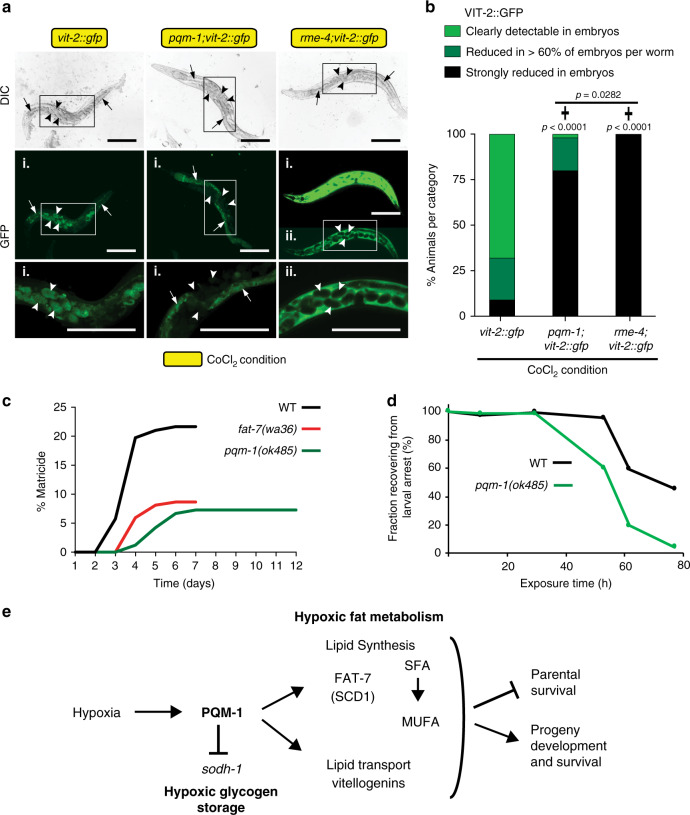
PQM-1 activity promotes vitellogenesis, matricide and progeny survival in hypoxic stress. **a**, **b** Effect of *pqm-1* loss on VIT-2::GFP levels in embryos (**a**) and quantification of VIT-2::GFP localization in embryos (**b**). Animals were synchronized at the L4 larval stage and exposed to 5 mM CoCl_2_ at the day 1 of adulthood stage for 24 h. i and ii indicate long and short exposure times, respectively. Arrows point to intestine; arrowheads point to embryos; scale bars: 200 µm. *vit-2::gfp* (*n* = 53), *pqm-1(ok485);vit-2::gfp* (*n* = 51), *rme-4(b1001);vit-2::gfp* (*n* = 32). Chi-square analysis was performed. (✝) Indicates mutant versus wild type *vit-2::gfp* expressing strain, three independent experiments. **c** Matricide analysis of *C. elegans* strains exposed to 5 mM CoCl_2_ at the L4 larval stage. WT (*n* = 157), *pqm-1(ok485)* (*n* = 165), *fat-7(wa36)* (*n* = 185). **d** Survival analysis of progeny originating from CoCl_2_ exposed hermaphrodites. The ability to exit and survive a developmental arrest caused by CoCl_2_ was analyzed after transferring arrested larvae to regular culture conditions. Chi-square analysis (**c**, **d**); **c** *p*-values for *pqm-1(ok485)* versus WT matricide timepoints: day 3 (*p* = 0.0018), day 4, day 5 (*p* < 0.0001), day 6 (*p* = 0.0001), day 7 (*p* = 0.0002); *fat-7(wa36)* versus WT: day 3 *(p* = 0.0010), day 4 (*p* = 0.0001), day 5 (*p* = 0.0006), day 6 (*p* = 0.0007), day 7 (*p* = 0.0007). **d** *p*-values for *pqm-1(ok485)* versus WT survival timepoints: 11 h, 30 h (ns = not significant), 53 h, 62 h, 77 h (*p* < 0.0001). Three independent experiments (**c**, **d**). **e** Model for PQM-1 as a regulator of hypoxic lipid and glycogen metabolism and survival. PQM-1 activity limits parental survival through promoting lipid biosynthesis via expression of the Δ9 stearoyl-CoA desaturase FAT-7 (SCD1 ortholog) and through positively regulating lipid transport via vitellogenin expression. Fat synthesis and transport support progeny formation and their survival in hypoxic stress conditions. PQM-1 activity represses sorbitol dehydrogenase (*sodh-1*) expression affecting hypoxic glycogen storage. SFA, saturated fatty acid: stearic acid [CH_3_(CH2)_16_COOH]; MUFA, mono-unsaturated fatty acid: oleic acid [CH_3_(CH_2_)_7_CH=CH(CH_2_)_7_COOH].

To further address whether *pqm-1* is required for vitellogenin expression in chemical hypoxia, we analyzed its effect on vitellogenin in developing embryos. We found that vitellogenin content was diminished (as shown by VIT-2::GFP translational fusion protein) in *pqm-1* embryos relative to wild-type embryos (Fig. 6a, b). VIT-2::GFP was already reduced in CoCl_2_-treated wild-type embryos compared to control conditions, but this downregulation was more pronounced in *pqm-1* mutants (Supplementary Fig. 6c). An *rme-4* mutant, which is deficient for vitellogenin uptake due to its reduced accumulation of the vitellogenin receptor on developing oocytes plasma membranes^35^, still maintained high levels of VIT-2::GFP, but excluded from the eggs (Fig. 6a, b and Supplementary Fig. 6c). Thus, vitellogenins are usually absorbed by oocytes, resulting in a depletion of the vitellogenin pool in hermaphrodites due to their downregulation under hypoxic stress. Our data suggest that PQM-1 positively regulates vitellogenin expression and promotes lipid reallocation to the progeny in hypoxic stress conditions.

Internal hatching of progeny in *C. elegans*, also known as “matricide,” has been proposed to be an adaptive response to stress or starvation^36^, enabling the parent to provide nutrients for larval development^29^ at the cost of the mother’s life. To assess the role of matricide in hypoxic survival, we determined the fraction of CoCl_2_-treated worms displaying internal hatching. *pqm-1* mutants reduced the occurrence of matricide relative to wild-type worms (Fig. 6c). Similarly, *fat-7* mutants showed reduced internal hatching under hypoxic stress (Fig. 6c). Together, these data suggest that the conversion of saturated fatty acids (SFAs) to mono-unsaturated fatty acids (MUFAs) during lipid biosynthesis is required to promote egg and progeny production in hypoxic stress.

Worms displaying intrauterine hatching were censored in CoCl_2_ based survival assays. To exclude the possibility that undetected matricide during the assay could contribute to survival effects, we treated worms with fluorodeoxyuridine (FUDR), an inhibitor of DNA synthesis, which suppresses internal hatching of progeny; *pqm-1(ok485)* mutants exposed to FUDR survived better than FUDR treated wild-type animals (Supplementary Fig. 6d). Therefore, these differences in survival are not due to reduction of matricide by *pqm-1* loss.

To address whether PQM-1 is also required for progeny function under hypoxia, we analyzed the ability of progeny of treated mothers to survive and recover from chemical hypoxia. Wild-type *C. elegans* larvae hatched from eggs that are laid by CoCl_2_-exposed hermaphrodites arrest as L1s if maintained on CoCl_2_. These L1-arrested progeny recover and develop into reproductive adults when they are transferred to regular culture conditions (Fig. 6d). However, progeny of CoCl_2_-treated *pqm-1* mothers are impaired in their ability to exit L1 larval arrest (Fig. 6d) and subsequently die, indicating that PQM-1 activity is essential for wild-type progeny to survive under hypoxic stress and subsequent recovery. Taken together, our results point to a balancing role for PQM-1 in hypoxic conditions, promoting progeny survival at the cost of somatic integrity of the parental generation (Fig. 6e).

## Discussion

Here we have found that the zinc finger transcription factor PQM-1 is a regulator of lipid metabolism and subsequent survival in hypoxic conditions, affecting both parental and progeny survival. Organisms metabolize energy sources through the process of respiration, and fatty acids and carbohydrates are the major energetic substrates for ATP production. The volume of oxygen consumed, and the volume of carbon dioxide produced (the “respiratory quotient”) depends on the fuel source. Molecules that are less oxidized, such as fatty acids, require more oxygen to be metabolized to CO_2_ and H_2_O than do fuel sources that are more oxidized, such as carbohydrates. Although lipid oxidation provides more ATP than carbohydrates, it also requires more oxygen per mole of ATP synthesized^37^. Therefore, glycolysis, especially anaerobic glycolysis, is an oxygen-saving but less effective process for energy production, in contrast to lipid catabolism, which consumes oxygen through oxidation of lipids in mitochondria^37^. When oxygen is abundant, fat metabolism is highly efficient and is thus the preferred catabolic pathway, but in hypoxic conditions, the relatively high rate of oxygen consumption can have deleterious effects on a tissue or an organism. Glycogen is the primary energy source for *C. elegans* in anoxia^18^.

We found that *pqm-1(ok485)* mutants maintain higher glycogen levels under chemical hypoxia conditions. Reduction of sorbitol dehydrogenase-1 (*sodh-1*), which is strongly upregulated in the absence of *pqm-1*, diminished the elevated glycogen content of *pqm-1(ok485)* mutants. SODH-1 enzymatic activity converts sorbitol, the sugar alcohol form of glucose, into fructose^21^. Sorbitol is metabolized to glycogen in diapause eggs of insects (*Bombyx mori*) depending on the state of the diapause^25^. SODH-1 activity is upregulated at the termination of diapause and metabolizes sorbitol to fructose, which is further converted to glycogen^25,26,38^. We anticipate that a similar sorbitol-to-glycogen metabolic pathway functions in *C. elegans*. Our data indicate that loss of *pqm-1* and thus elevated *sodh-1* expression is associated with increased glycogen storage, while loss of *pqm-1* resulted in a reduction of *fat-7* desaturase expression and diminished lipid levels under hypoxic stress.

The enzymatic reaction that FAT-7 carries out, desaturation of stearic acid to oleic acid, requires molecular oxygen^39^, and our data suggest that basal and maximal oxygen consumption rates are decreased in both *pqm-1* and *fat-7* mutants. A functional link between the desaturation of lipids and respiration has been previously established in plants^40^, through a deficiency in an ω−6-oleate desaturase, which regulates lipid metabolism and respiration. Similarly, we found that basal and maximal oxygen consumption rates are decreased in both *pqm-1* and *fat-7* mutants. Our data suggest that PQM-1 might act as a metabolic gatekeeper in hypoxia, repressing *sodh-1* expression and glycogen levels, while promoting the expression of the stearoyl-CoA desaturase FAT-7. FAT-7 activity, in turn, regulates lipid biosynthesis and lipid desaturation, which is required for respiratory activity and oxygen consumption.

The suppression of lipid metabolism in a hypoxic tissue that largely relies on carbohydrates (glucose) as an energy source is demonstrated during cardiac development^41^. The developing embryonic heart is exposed to hypoxic conditions, but is highly protected against hypoxic stress through the reduction of lipid metabolic processes^42^. Downregulation of fat metabolism by the activity of the basic helix-loop-helix transcription factor HAND1 decreases both basal and maximal OCRs. The decreased oxygen consumption rate in cardiomyocytes provides a protective metabolic strategy for tissue development under low oxygen tension. Inhibition of cellular lipid metabolism by etomoxir, a carnitine palmitoyltransferase antagonist that prevents mitochondrial long-chain fatty acid import, also decreases OCR and protects against myocardial ischemia^43,44^; downregulation of lipid metabolism and OCRs resulted in a shift of energy metabolism to glycolysis.

Why has a PQM-1-mediated mechanism to boost hypoxic fat levels evolved, if this seems to have negative implications for survival under hypoxic stress? We hypothesize that PQM-1 positively regulates intestinal fat production and transportation to oocytes to fuel reproduction in hypoxic animals. Such a strategy might enable some progeny to escape hypoxic stress conditions, thereby ensuring survival of the population. An investment in reproduction is beneficial for the population, even if detrimental to the individual mother. A similar trade-off between viability and fecundity has been previously described when *C. elegans* is exposed to nutrient-poor and oxidative stress environments^29^. During reproduction, somatic resources, particularly lipids, are reallocated to the germline. Fat is produced in the intestine and the hypodermis and transported by the actions of vitellogenins^45^, which assemble with transport lipids in the form of yolk to shuttle fat from the intestine to the developing oocytes. When resources are limited, lipid reallocation appears to promote fecundity at the cost of somatic integrity of the parental generation. We found that vitellogenins were downregulated in *pqm-1* mutants upon hypoxic stress (Fig. 5e, f and Supplementary Fig. 5c, Supplementary Data 2); thus, vitellogenin genes are normally positively regulated by PQM-1 upon hypoxic stress, to facilitate lipid transport from the mother’s intestine into developing eggs and to support progeny survival. The transcriptional properties of PQM-1 appear to be dependent on additional transcription factors such as CEH-60, which functions as a key regulator for the expression of vitellogenins along with UNC-62^32,34,46,47^. Previously, it was demonstrated that PQM-1 suppresses vitellogenin expression and acts as a downstream transcriptional effector of TORC2 signaling^48^. However, these studies were performed under regular, normoxic growth conditions, rather than hypoxic stress. Thus, it appears that PQM-1 controls vitellogenin expression depending on environmental conditions, which might affect the promoter context of its target genes resulting in different transcriptional responses under stress versus standard growth conditions.

Taken together, PQM-1 activity under hypoxic stress increases lipid levels by positively regulating fatty acid synthesis via *fat-7* expression, and by promoting reallocation of fat to embryos through vitellogenin expression. This, in turn, boosts lipid-dependent embryonic development inside the worm’s uterus (Figs. 5a, b; 6a, b) and promotes progeny survival in a hypoxic environment (Fig. 6d). Somatic allocation of limited lipid resources into the promotion of reproduction during hypoxic stress is detrimental for individual viability, but may increase the chance of species survival through investment in the next generation. Because lipid and carbohydrate-related pathways are well conserved across species, deciphering mechanisms implicated in metabolic remodeling in *C. elegans* will be beneficial for the development of treatment strategies to treat cancer and age-related diseases in humans.

## Methods

### *C. elegans* genetics

All strains were cultured using standard methods^49^. In all experiments, N2 is wild type.

### Strains

CQ200 (*pqm-1(ok485*);*daf-2(e1370)*);

CQ528 (*pqm-1(ok485)*);

CF1041 (*daf-2(e1370*));

CQ565 (*daf-16(mu86);pqm-1(ok485)*)*;*

DMS303 (*nIs590[Pfat-7::fat-7::GFP* + *lin-15(+)]*)*;*

BR8808 (*daf-2(e1370*);*nIs590[Pfat-7::fat-7::GFP* + *lin-15(+)]*)*;*

BR8807 (*pqm-1(ok485*);*daf-2(e1370)*;*nIs590[Pfat-7::fat-7::GFP* + *lin-15(+)]*)*;*

CQ609 (*pqm-1(ok485*);*(nIs590[Pfat-7::fat-7::GFP* + *lin-15(+)]*)*;*

BX153 (*fat-7(wa36)*);

BR8610 (*sodh-1(ok2799)*);

BR8611 (*pqm-1(ok485);sodh-1(ok2799)*);

CF1038 (*daf-16(mu86)*);

CF2124 (*muIs139[dod-11p::RFP(NLS)* + *rol-6(su1006)]*);

BR8809 (*daf-16(mu86)*;*muIs139[dod-11p::RFP(NLS)* + *rol-6(su1006)]*);

RT130 (*pwIs23[vit-2::GFP]*);

BR8724 (*pqm-1(ok485);pwIs23[vit-2::GFP]*);

RT362 (*rme-4(b1001);pwIs32[vit-2::GFP]*); OP201 (*unc-119(tm4063);wgIs201[pqm-1::TY1::EGFP::3xFLAG(92C12) + unc-119(+)]*).

All animals were synchronized at the L4 larval stage for adult analyses, as *pqm-1* mutants develop slower than do wild-type animals^15^.

### Survival analysis

Synchronized L4 larvae were picked onto plates to eliminate any contribution of delayed development into survival analysis. Worm were grown for several generations on 20 °C without starvation. The log-rank (Mantel–Cox) method was used to test the null hypothesis (two-sided hypothesis test) in Kaplan–Meier survival analysis, as previously described^50^, and evaluated using Prism survival analysis software. All survival experiments were carried out at 20 °C; *n* ≥ 60 per strain/trial.

### Cobalt chloride-based survival assays

A 200 mM cobalt chloride (CoCl_2_) stock solution (Cobalt(II) chloride hexahydrate, Sigma-Aldrich) was prepared, and filter sterilized using a 0.22 µm filter as described previously^8^. OP50 bacteria (60 µl bacteria solution) were seeded in the center of 6 cm NG plates previously dried for 3–4 days at room temperature. OP50 bacteria were grown on plates for 18–20 h at room temperature. To prepare CoCl_2_ plates, 250 µl of the 200 mM CoCl_2_ stock solution was added to 6 cm NG plates (contain ~10 ml NG agar) with seeded, over-night grown OP50 bacteria, which results in a 5 mM final concentration of CoCl_2_. The CoCl_2_ solution was immediately distributed equally over the surface of the plate with a spreader (carefully, not to remove seeded OP50; a thin OP50 layer can result in increased bagging of worms during the assay). The CoCl_2_ solution was completely taken up by the plate resulting in dry plates before worms were added. A thin ring of 100% glycerol was added along the plastic wall of the plate to reduce the number of worms crawling off the agar. Plates were incubated with lids facing up, which can reduce the number of worms crawling off CoCl_2_ containing plates. To obtain synchronized worm cultures, 75–180 L4 larvae were picked for each strain, grown for 3 h on OP50 bacteria and added to freshly prepared CoCl_2_ plates (3 h-post-L4 picking, corresponds to first day in survival assay). Droplets of moisture were removed from the inside of the plate’s plastic wall using a sterilized filter paper. Thus, the number of worms escaping into water droplets is reduced during survival assays. Worms displaying intrauterine hatching were censored. Intrauterine hatching is difficult to control in CoCl_2_-based assays and might vary between experiments. The log-rank (Mantel–Cox) method was used to test the null hypothesis (two-sided hypothesis test) in Kaplan–Meier survival analysis, as previously described^50^, and evaluated using Prism survival analysis software.

### Cobalt chloride-based matricide assays

L4-synchronized worms and CoCl_2_-containing plates were prepared as described above for survival assays. Worms are exposed to CoCl_2_ at the L4 stage (Fig. 6c) or the day 1 of adulthood stage (Fig. 4d). Exposure of worms at the L4 stage results in reduced matricide rates over time (e.g. ~10–25% for wild type worms), enabling the calculation of matricide curves (Fig. 6c), whereas challenging day 1 adult worms with CoCl_2_ causes already higher rates of internal hatching after 2 days of CoCl_2_ exposure (Fig. 4d). In experiments for Fig. 4d worms were immediately seeded to CoCl_2_-containing OP50 plates after CoCl_2_ was taken up by the plate.

### Oil Red O-based lipid staining and quantification

Worms were grown for several generations on 20 °C without starvation. For lipid staining experiments, L4-synchronized worms were grown to day 1 of adulthood. OP50 Plates were prepared as described above. CoCl_2_ solution was added to plates (2.5 mM final concentration). 0.5–1 h after adding CoCl_2_ to plates seeded with OP50 bacteria, worms were transferred to CoCl_2_ plates and exposed to CoCl_2_ conditions for 40–52 hr. A 0.5% Oil Red O stock solution was prepared in high-quality 100% isopropanol as described previously^51^. For staining of worms, the stock solution was diluted to 60% with sterile water, incubated on a rocking platform at room temperature overnight and filtered through a 0.45 µm filter. Worms were resuspended in 500 µl 60% isopropanol for fixation. Isopropanol was aspirated, 500 µl of freshly filtered Oil Red O working solution was added and worm strains were incubated in a Thermomixer at 25 °C using mild agitation (550 rpm) for 16 h. Animals were washed three times with 500 µl of 0.01% Triton X-100 in M9 buffer and stored at 4 °C followed by imaging on a Nikon Eclipse Ti microscope (20x objective) or an Axioplan 2 Imaging microscope (Carl Zeiss AG, 10x objective for worms, ×20 objective for eggs). For quantification of the lipid content of eggs based on Oil Red O staining, eggs were dissected out of Oil Red O-stained worms followed by imaging as describe above (20x objective). Oil Red O quantification was performed as previously described^52^. In brief, color images were split into RGB monochromatic images in Image J (Fiji). The Oil Red O intensity was determined by calculating the mean gray value within a worm region or within an egg area (Intensity of the blue channel was used as the signal, adjusted by the intensity in the Red channel as the background).

### RNA collection and microarray hybridization and analysis

RNA was purified from hypochlorite-synchronized populations of early day 1 adults using Trizol (Gibco) and the RNeasy kit (Qiagen)^14,53^. Using the two-color microarray-based gene expression analysis kit from Agilent Technologies, cRNA was synthesized and linearly amplified from 100 ng of total RNA, labeled with Cy3 and Cy5-CTP dyes (Amersham), fragmented and hybridized overnight on Agilent 4 × 44K *C. elegans* arrays at 60 °C. Three biological replicates of CoCl_2_-treated *pqm-1(ok435)* versus CoCl_2_-treated wild type were compared to untreated *pqm-1(ok435)* versus untreated wild type_._ In addition, CoCl_2_-treated *pqm-1(ok435)* versus untreated *pqm-1(ok435)* were compared to CoCl_2_-treated WT versus untreated WT. Significant differentially-expressed gene sets were identified using one or two-class SAM^54^. Gene ontology analysis (GO) was performed utilizing DAVID (Database for Annotation, Visualization and Integrated Discovery)^55^ and g:Profiler^56^. GO categories were visualized with REVIGO (Reduce and Visualize Gene Ontology)^57^. The accession number for the microarray experiments reported in this paper is: GSE139562 (NCBI Gene Expression Omnibus (GEO) repository).

### Quantitative RT–PCR

Total RNA was isolated as described above for microarray analysis. Quantitative RT**–**PCR was performed as described previously^58^. SybrGreen real-time PCR experiments were performed with a 1:8 dilution of cDNA using a LightCycler 96 Instrument (Roche Life Science) following the manufacturer’s instructions. Data were analyzed with the standard curve method using the geometric mean of *cdc-42*, *pmp-3* and Y45F10D.4 as an endogenous control^59^. Primers for the quantification of vitellogenin and *ceh-60* expression have been described previously^34^. Additional primers utilized for real-time PCR of *sodh-1, fat-7, and unc-62* are listed in Supplementary Table 1.

### Glycogen staining

Worm strains were exposed at the L4 larval stage to 5 mM CoCl_2_ for 4–5 days. Differences in glycogen levels between strains were more pronounced when worms were exposed to CoCl_2_ for a longer time period such as 4–5 days. To reduce lethality or matricide of worms for a prolonged incubation with CoCl_2_, the CoCl_2_ solution was added to OP50 seeded plates 0.5 h to 1 h before worms were added. Worms displaying internal hatching during prolonged CoCl_2_ exposure were not used for the Glycogen straining. Following CoCl_2_ exposure, two worm strains were pairwise compared by picking individuals into an M9 droplet on an agar pad (two small agar pads were placed next to each other on the same slide). As soon as the M9 droplets were largely evaporated, the pads were inverted over the opening of a 50 g bottle of iodine crystal chips (Sigma, St. Louis, MO) for 90 s^18^. After the iodine stained color of the agar pads disappeared (non-specific staining), worms (about 10–15 worms per treatment) were immediately imaged on a Nikon Eclipse Ti microscope (×20 objective) or an Axioplan 2 Imaging microscope (Carl Zeiss AG, 10x objective). Image J (Fiji) was used to determine the mean intensity of iodine staining within a worm region after the background was subtracted.

### Measurement of oxygen consumption rate (OCR)

OCR measurement was carried out as previously described^60^. Worms were synchronized at the L4 stage and OCR measurement was performed at early adulthood (day 1 of adulthood stage). Approximately 15–20 animals were pipetted into each well of a Seahorse XF96 utility plate. Six replicates per strain were used (6 biological repeats). The Seahorse program was set up in a way that each oxygen consumption measurement consisted of a two-minute mix cycle, followed by a one-minute wait period (to allow worms to settle), and finally a 2-min interval for measurement of oxygen levels. OCRs were normalized by worm number and worm body area. To determine the basal OCR, we averaged the first tree measurements. The uncoupler FCCP was injected twice (10 µM each injection) followed by sodium acid injection (40 mM). The first two measurements after FCCP injection were variable. Therefore, we averaged measurements 3–10 after FCCP injection to obtain the maximal OCR.

### Hypoxic incubation

Survival analysis of worms in a hypoxic glove box (Ruskinn Invivo 400 Hypoxic Workstation) were performed as previously published^17^. Worms at the day 1 of adulthood stage (synchronized at the L4 stage) were exposed in 200 µl M9 buffer in a 1.5 ml tube to <0.3% O_2_ (balanced with nitrogen) for 16 hr at 26 °C followed by an 8 h or one-day recovery period on OP50 plates in room air at 20 °C. Oxygen levels in the hypoxic glove box were additionally monitored using a Greisinger GMH 3692 oxygen meter with a Greisinger GGO 370 oxygen sensor.

### L1 arrest-based survival assay of progeny

L1 larvae were hatched from eggs laid on OP50 plates containing 5 mM CoCl_2_. Larvae did not develop further and arrested when exposed to CoCl_2_. 60–180 arrested larvae per strain were transferred every day to fresh OP50 plates without CoCl_2_ and analyzed for their ability to exit and survive the larval arrest and develop to adulthood.

### RNAi treatment

RNAi-treated worm strains were fed *E. coli* (OP50(xu363))^61^ containing an empty-vector construct or a construct expressing double-stranded RNA (dsRNA) against the gene of interest. *pqm-1(ok485)*-mediated survival extension in CoCl_2_ assays is dependent on the OP50 bacterial background. All RNAi-mediated knock down experiments were performed in the OP50(xu363) bacterial background, except knock downs of *ceh-60* and *unc-62* for RT-qPCR experiments were performed in the HT115 bacterial background, because the *ceh-60* construct inhibited growth of OP50(xu363) bacteria.

### Statistics and reproducibility

All data in this article are expressed as mean ± standard deviation (SD) unless otherwise noted. Two-tailed *t*- test analysis, two-way ANOVA analysis, and two-sided Chi-square analysis were performed to compare different groups in this study (without adjustments for multiple comparisons). Confidence intervals of 95% were chosen for two-tailed *t* -test and Chi-square analysis. Probability values below 0.05 were considered statistically significant. Kaplan–Meier analysis with log-rank (Mantel–Cox) method was applied using a two-sided hypothesis test (with Prism) to compare survival curves of different groups. Three independent experiments were performed unless otherwise noted, and representative results were shown. RT-qPCR experiments were performed once using three to four biological replicates to validate microarray and reporter-based analysis. Significant differentially-expressed gene sets originating from microarrays were identified by one or two-class SAM^54^. SAM uses two-sided statistical testing and reports q-values, which are false discovery rates (multiple comparisons are performed).

## Supplementary information

Supplementary Information

Description of Additional Supplementary Information

Supplementary Data 1

Supplementary Data 2

Supplementary Data 3

Supplementary Data 4

## Data Availability

Microarray datasets generated and analyzed during the current study are available in the NCBI Gene Expression Omnibus (GEO) repository under the GEO accession number GSE139562. All other data that support the findings of this study are available from the corresponding authors upon request. Following databases/analysis tools have been used for data analysis in this study: DAVID, Database for Annotation, Visualization and Integrated Discovery [https://david.ncifcrf.gov/]; g:Profiler [https://biit.cs.ut.ee/gprofiler/gost]; REVIGO, Reduce and Visualize Gene Ontology [http://revigo.irb.hr/]; SAM, Significance Analysis of Microarrays [https://statweb.stanford.edu/~tibs/SAM/] and Princeton University MicroArray database, PUMAdb [https://puma.princeton.edu/about.shtml]. Source data are provided with this paper.

## Source data

Source Data
