# Author Correction: PQM-1 controls hypoxic survival via regulation of lipid metabolism

**DOI:** 10.1038/s41467-020-19868-6

**Published:** 2020-11-20

**Authors:** Thomas Heimbucher, Julian Hog, Piyush Gupta, Coleen T. Murphy

**Affiliations:** 1 Lewis-Sigler Institute for Integrative Genomics, Princeton University, Princeton, NJ 08544 USA; 2 Department of Molecular Biology, Princeton University, Princeton, NJ 08544 USA; 3 Bioinformatics and Molecular Genetics, Faculty of Biology, University of Freiburg, Freiburg, 79104 Baden-Wuerttemberg, Germany

**Keywords:** Development, Gene expression, Molecular biology

Correction to: *Nature Communications* 10.1038/s41467-020-18369-w, published online 2 October 2020.

The original version of this Article omitted the following from the Acknowledgements:

Research of T.H. and P.G. in the Baumeister lab was supported by the Deutsche Forschungsgemeinschaft DFG (German Research Foundation under Germany’s Excellence Strategy EXC 294 and EXC-2189-Projektnummer 390939984; CRC850-Project ID A06 and CRC1381-Project ID B09 to Ralf Baumeister).

This has now been corrected both the PDF and HTML versions of the Article.

