# Supplementary material for: PQM-1 controls hypoxic survival via regulation of lipid metabolism
Source: Nat Commun. 2020 Oct 2;11:4627. doi: 10.1038/s41467-020-18369-w (PMC7532158; doi:10.1038/s41467-020-18369-w)
Supplement: Supplementary file 1 — Supplementary Information [file 41467_2020_18369_MOESM1_ESM.pdf]

Supplementary Information

PQM-1 controls hypoxic survival via regulation of lipid metabolism

Heimbucher et al.

Supplementary Figure 1: *pqm-1* inactivation increases hypoxic survival independently of *daf-16*

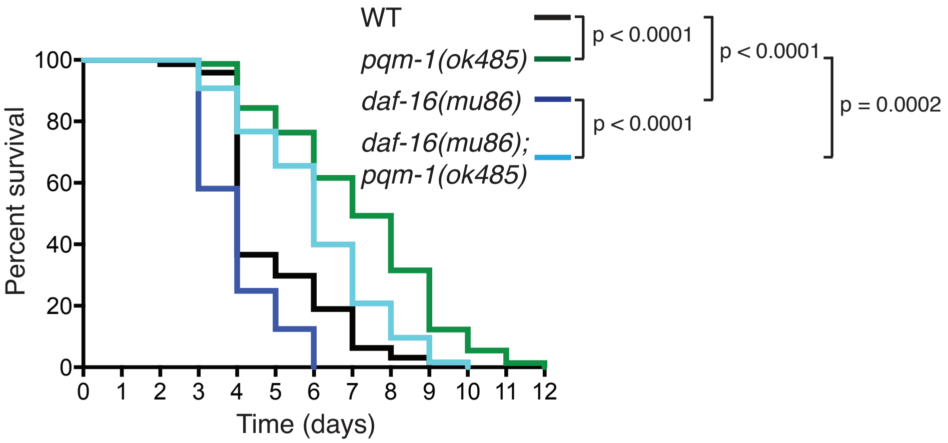

Survival analysis of animals exposed to 5 mM $CoCl_2$ at the L4 larval stage. WT (N2) (n = 58), *pqm-1(ok485)* (n = 74), *daf-16(mu86)* (n = 51), *daf-16(mu86);pqm-1(ok485)* (n = 63). Log-rank analysis (two-sided), three independent repeats. Source data are provided as a Source Data file for Supplementary Fig. 1.

Supplementary Figure 2: Gene expression changes and Gene Ontology analysis of $CoCl_2$ and *pqm-1*- regulated genes

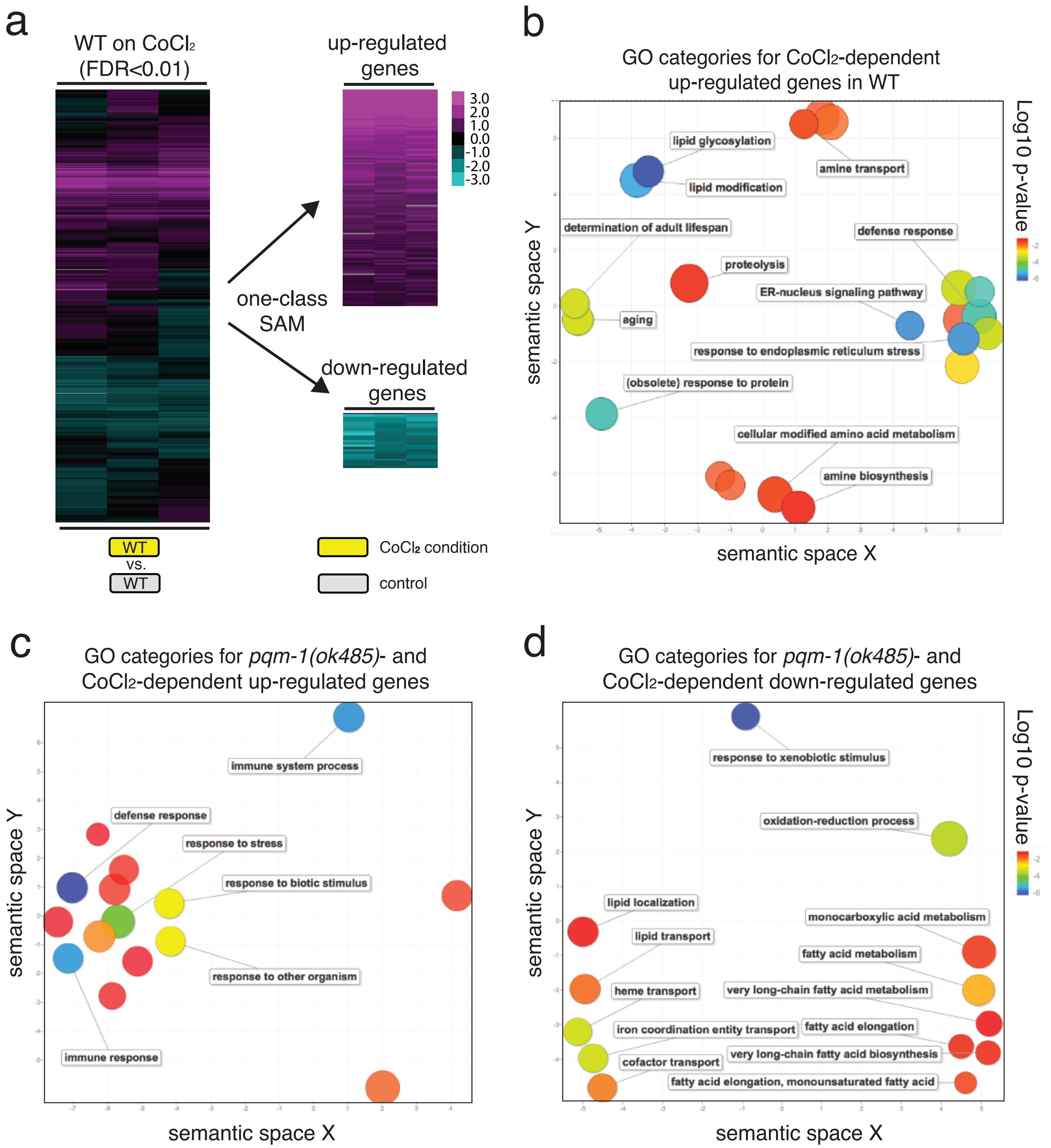

a, b) Gene expression (a) and Gene Ontology (GO)-based analysis (b) of $CoCl_2$-regulated target genes in wild-type (WT) animals. One-class SAM (WT +$CoCl_2$ versus WT -$CoCl_2$) identified 366 upregulated and 56 downregulated genes (Supplementary Table 1). c, d) GO term enrichment analysis (biological processes) of genes up-regulated (c) and downregulated (d) in $CoCl_2$-treated *pqm-1(ok485)* mutants versus $CoCl_2$-treated WT (Supplementary Table 2). GO term enrichment analysis was performed using g:Profiler and plotted by REVIGO. The size of the circles is indicative of the number of genes annotated with that GO term; circles are color-coded according to significance (blue color indicates highest significance).

Supplementary Figure 3: GO term enrichment analysis and *sodh-1* expression study

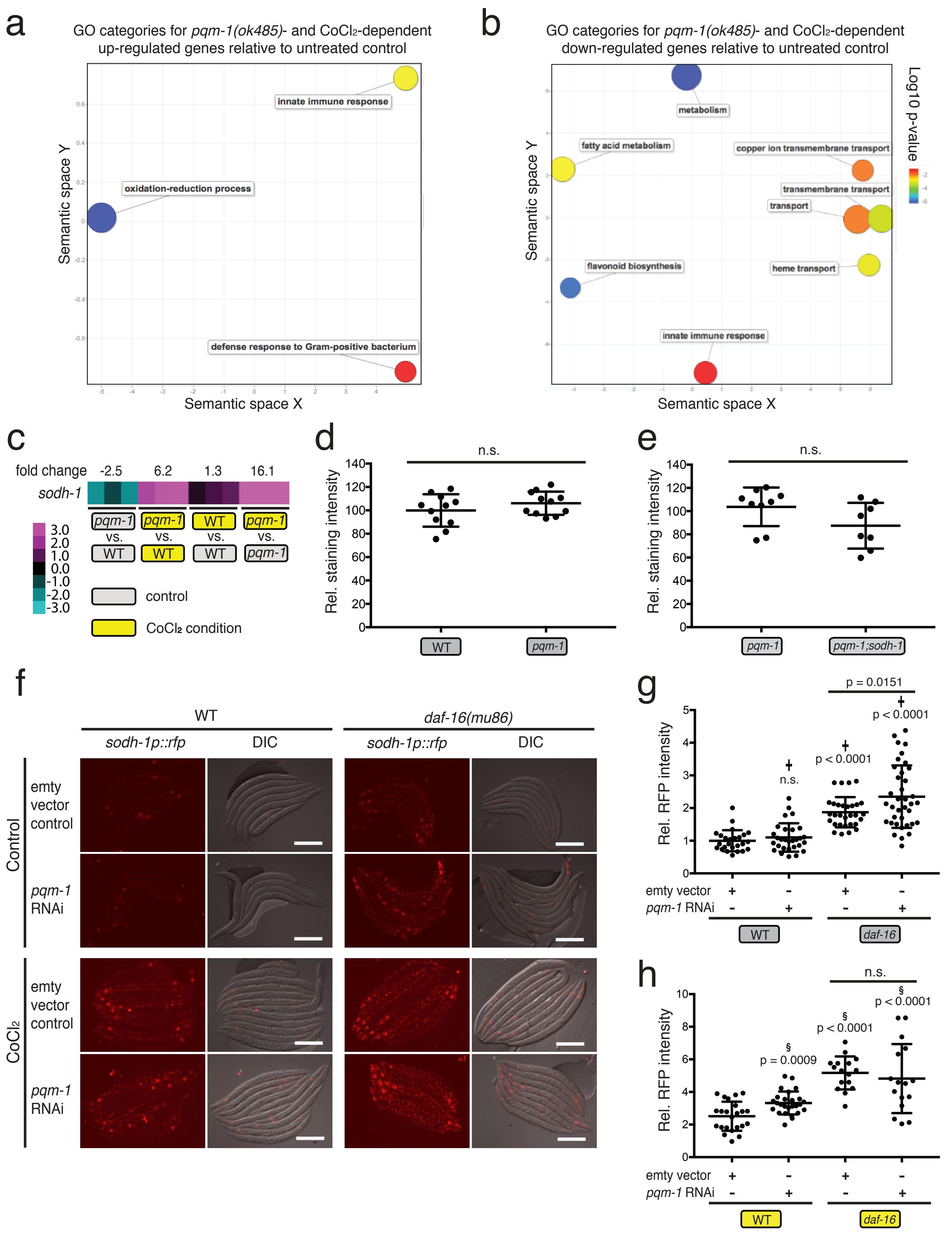

a, b) GO term enrichment analysis (biological processes) of upregulated genes (a) and downregulated genes (b) identified with two-class SAM in *pqm-1(ok485)* mutants challenged with $CoCl_2$ versus wild type (WT) with $CoCl_2$ compared to *pqm-1(ok485)* control versus WT control conditions (Fig. 2b, c). GO analysis was performed using DAVID and plotted by REVIGO. The size of the circles is indicative of the number of genes annotated with that GO term; circles are color-coded according to significance (blue color indicates highest significance). c) *pqm-1*- and $CoCl_2$-dependent expression changes for endogenous *sodh-1* based on heat maps originating from microarrays (Fig. 2b, Supplementary Table 3). Animals were exposed to 5 mM $CoCl_2$ for 6 hr at early adulthood. d, e) Quantification of glycogen levels determined by iodine vapor staining (d) in wild type (n = 11), *pqm-1(ok485)* (n = 11), and (e) in *pqm-1(ok485)* (n = 9) and *pqm-1(ok485);sodh-1(ok2799)* mutants (n = 8) for control conditions. Quantification values were normalized to mean value of WT control worms without $CoCl_2$.Two-tailed t-test, mean +/- SD; ns = not significant, three independent experiments. f-h) *sodh-1p::rfp* reporter activity in control conditions and after 12 hr of 5 mM $CoCl_2$ exposure at a late larval (L4) stage; (g, h) quantification of reporter activity in (f). Quantification values were normalized to mean value of WT empty vector control without $CoCl_2$. Two-tailed *t*-test (g, h), mean +/- SD, (**+**) indicates statistical significance for conditions without $CoCl_2$ versus wild-type empty vector control without $CoCl_2$ (g). WT (N2) (n = 25) and *daf-16(mu86)* (n = 30) on empty vector; WT (N2) (n = 29) and *daf-16(mu86)* (n = 37) on *pqm-1* RNAi; (§) indicates statistical significance for conditions with $CoCl_2$ versus wild-type empty vector control exposed to $CoCl_2$ (h). WT (N2) (n = 23) and *daf-16(mu86)* (n = 16) on empty vector; WT (N2) (n = 26) and *daf-16(mu86)* (n = 16) on *pqm-1* RNAi; ns = not significant; scale bars: 200 μm. Three independent experiments were performed. Source data are provided as a Source Data file for Supplementary Fig. 3d, e, g, h.

Supplementary Figure 4: *fat-7* analysis

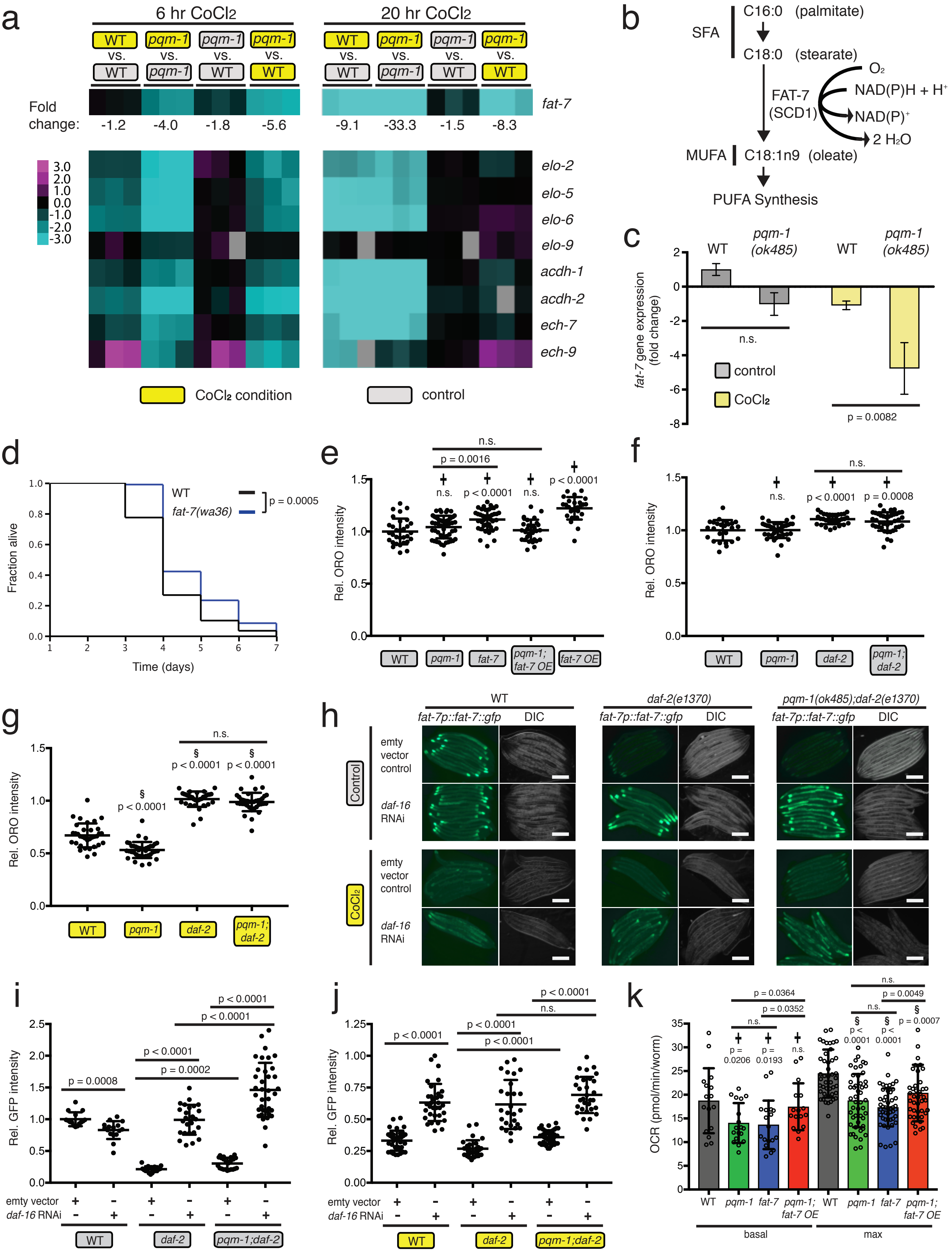

a) Gene expression analysis-based heat maps indicating a PQM-1 and $CoCl_2$-dependent expression of *fat-7* upon 6 hr and 20 hr exposure of animals to 5 mM $CoCl_2$ at the early adulthood stage. Additional indicated lipid regulators down-regulated at 6 hr $CoCl_2$ exposure are not significantly down-regulated in a *pqm-1* dependent way at 20 hr $CoCl_2$ exposure (in critical 4th condition of heat map). Heat maps depict individual genes (rows), with columns representing individual microarrays. b) The Δ9 stearoyl-CoA desaturase (SCD1) ortholog FAT-7 desaturates stearate to oleate and requires oxygen as an electron acceptor. SFA, saturated fatty acid; MUFA, mono-unsaturated fatty acid; PUFA, poly-unsaturated fatty acid; c) *pqm-1*- and $CoCl_2$-dependent regulation of endogenous *fat-7* determined by RT-qPCR (n = 3 biological replicates) following 5 mM $CoCl_2$ exposure for 6 hr at early adulthood. Two-tailed *t*-test, mean +/- SD, ns = not significant. d) Survival analysis of *fat-7* loss-of-function mutants exposed to 5 mM $CoCl_2$ at the L4 larval stage. WT (n = 65), *fat-7(wa36)* (n = 82), log-rank analysis (two-sided), three independent repeats. e) Whole worm quantification of fat levels for control conditions based on the Oil Red O staining for Fig. 4e, f. WT (N2) (n = 31), *pqm-1(ok485)* (n = 51), *fat-7(wa36)* (n = 39), *pqm-1(ok485);fat-7* OE (n = 29), *fat-7* OE (n = 26); three independent experiments. f, g) Oil Red O based whole worm quantification of fat levels for control conditions (f) and worms exposed to 2.5 mM $CoCl_2$ at the day 1 of adulthood stage for 52 hr (g). Quantification values were normalized to mean value of WT control without $CoCl_2$ (e-g). Two-tailed *t*-test (e-g), mean +/- SD, (**+**) indicates mutant versus wild type strains for control conditions; WT (N2) (n = 25), *pqm-1(ok485)* (n = 36), *daf-2(e1370)* (n = 33), *pqm-1(ok485);daf-2(e1370)* (n = 39). (§) indicates mutant versus wild type strains for $CoCl_2$ conditions; WT (N2) (n = 34), *pqm-1(ok485)* (n = 39), *daf-2(e1370)* (n = 34), *pqm-1(ok485);daf-2(e1370)* (n = 37), ns = not significant; two independent experiments. h-j) *fat-7p::fat-7::gfp* reporter activity in control conditions and after 25 hr of 5 mM $CoCl_2$ exposure at a late larval (L4) stage; (i, j) quantification of reporter activity in (h). Quantification values were normalized to mean value of WT empty vector control without $CoCl_2$ for control condition (i) and $CoCl_2$ exposure (j). (i) WT (n = 15), *daf-2(e1370)* (n = 20), *pqm-1(ok485);daf-2(e1370)* (n = 23) on empty vector. WT (n = 16), *daf-2(e1370)* (n = 26), *pqm-1(ok485);daf-2(e1370)* (n = 36) on *daf-16* RNAi. (j) WT (n = 37), *daf-2(e1370)* (n = 31), *pqm-1(ok485);daf-2(e1370)* (n = 44) on empty vector. WT (n = 33), *daf-2(e1370)* (n = 27), *pqm-1(ok485);daf-2(e1370)* (n = 30) on *daf-16* RNAi. Two-tailed *t*-test (i, j), mean +/- SD, ns = not significant; scale bars: 200 μm. Three independent experiments were performed. k) Measurement of oxygen consumption rates for a rescue of *fat-7* expression in a *pqm-1(ok485)* mutant. The uncoupler FCCP was injected twice (10 μM each injection) to obtain maximal oxygen consumption rates. For each strain six biological repeats, each consisting of 15-20 worms, have been repeatedly measured to obtain OCR values (source data file). Two-tailed *t*-test, mean +/- SD. (**+**) indicates statistical significance for mutant versus wild type strains for basal OCR. (§) indicates statistical significance for mutant versus wild type strains for maximal OCR, ns = not significant. Animals were synchronized at the L4 larval stage for adult analyses (d-k). Source data are provided as a Source Data file for Supplementary Fig. 4c-g, i-k.

Supplementary Figure 5: PQM-1 promotes embryonic lipid levels and affects CEH-60-dependent hypoxic vitellogenin expression

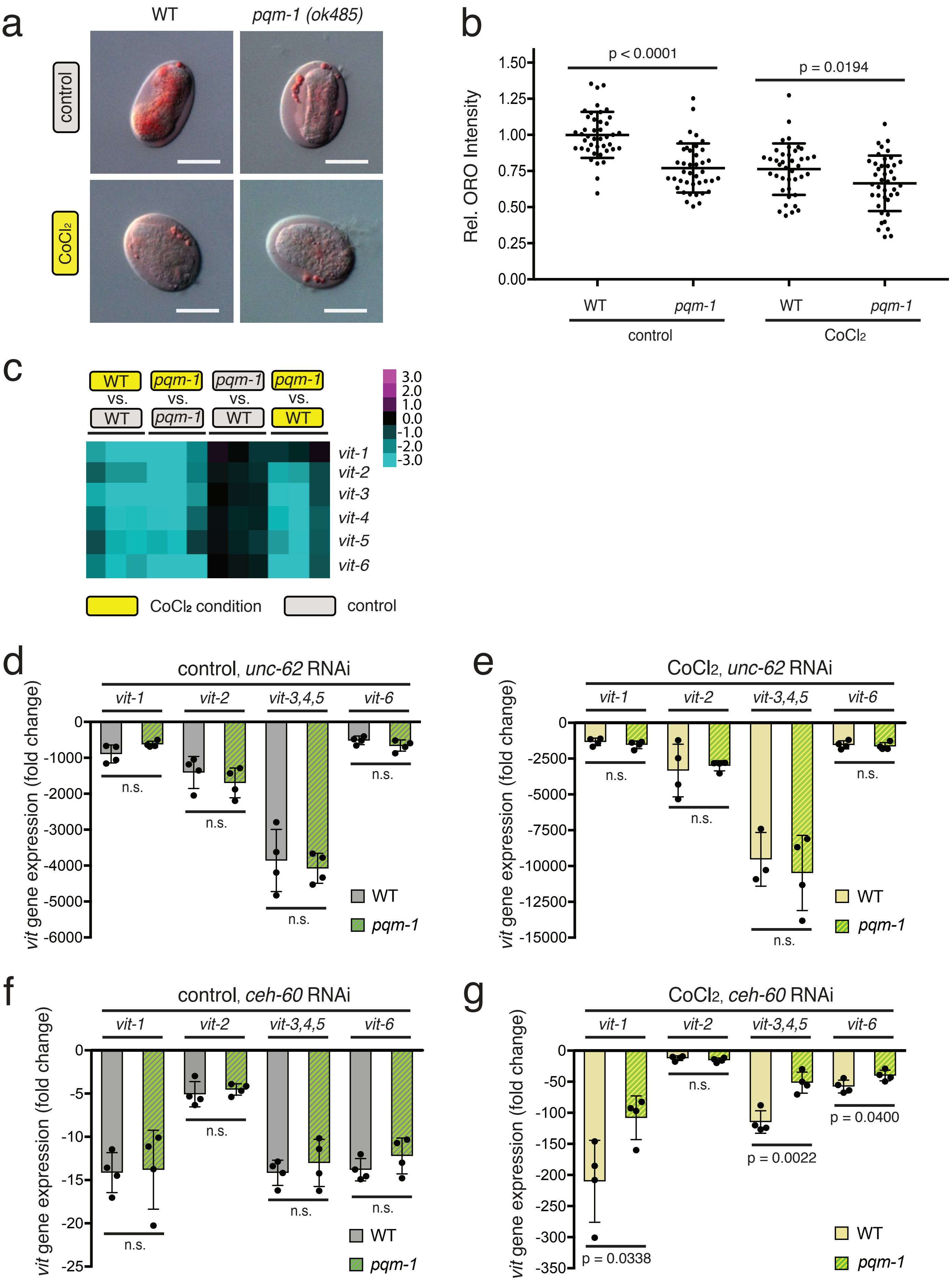

a, b) Lipid content of eggs (a) dissected from Oil Red O-stained hermaphrodites challenged with 2.5 mM $CoCl_2$ at the day 1 of adulthood stage for 52 hr and quantification of fat levels in eggs (b). WT (N2) (n = 42), *pqm-1(ok485)* (n = 40) in control condition; WT (N2) (n = 40), *pqm-1(ok485)* (n = 41) in $CoCl_2$ condition; Scale bars: 25 μm; two-tailed *t*-test, mean +/- SD, two independent experiments. c) Gene expression analysis-based heat maps indicating a PQM-1 and $CoCl_2$-dependent expression of vitellogenins. Heat maps depict individual vitellogenin genes (rows) with columns representing individual microarrays. Animals were exposed to 5 mM $CoCl_2$ for 20 hr at early adulthood. d, e) *pqm-1*- and $CoCl_2$-dependent regulation of endogenous vitellogenins in *unc-62* RNAi treated animals determined by RT-qPCR. f, g) *pqm-1*- and $CoCl_2$-dependent regulation of endogenous vitellogenins in *ceh-60* RNAi treated animals determined by RT-qPCR. Values for vitellogenin expression were normalized to wild-type empty vector control without $CoCl_2$ (d-g). Animals were exposed to 5 mM $CoCl_2$ for 20 hr at early adulthood (d-g). Three to four biological replicates were used. Two-tailed t-test (d-g), ns = not significant. Source data are provided as a Source Data file for Supplementary Fig. 5b, d-g.

Supplementary Figure 6: PQM-1 promotes *unc-62* expression and embryonic VIT-2 levels in hypoxic stress

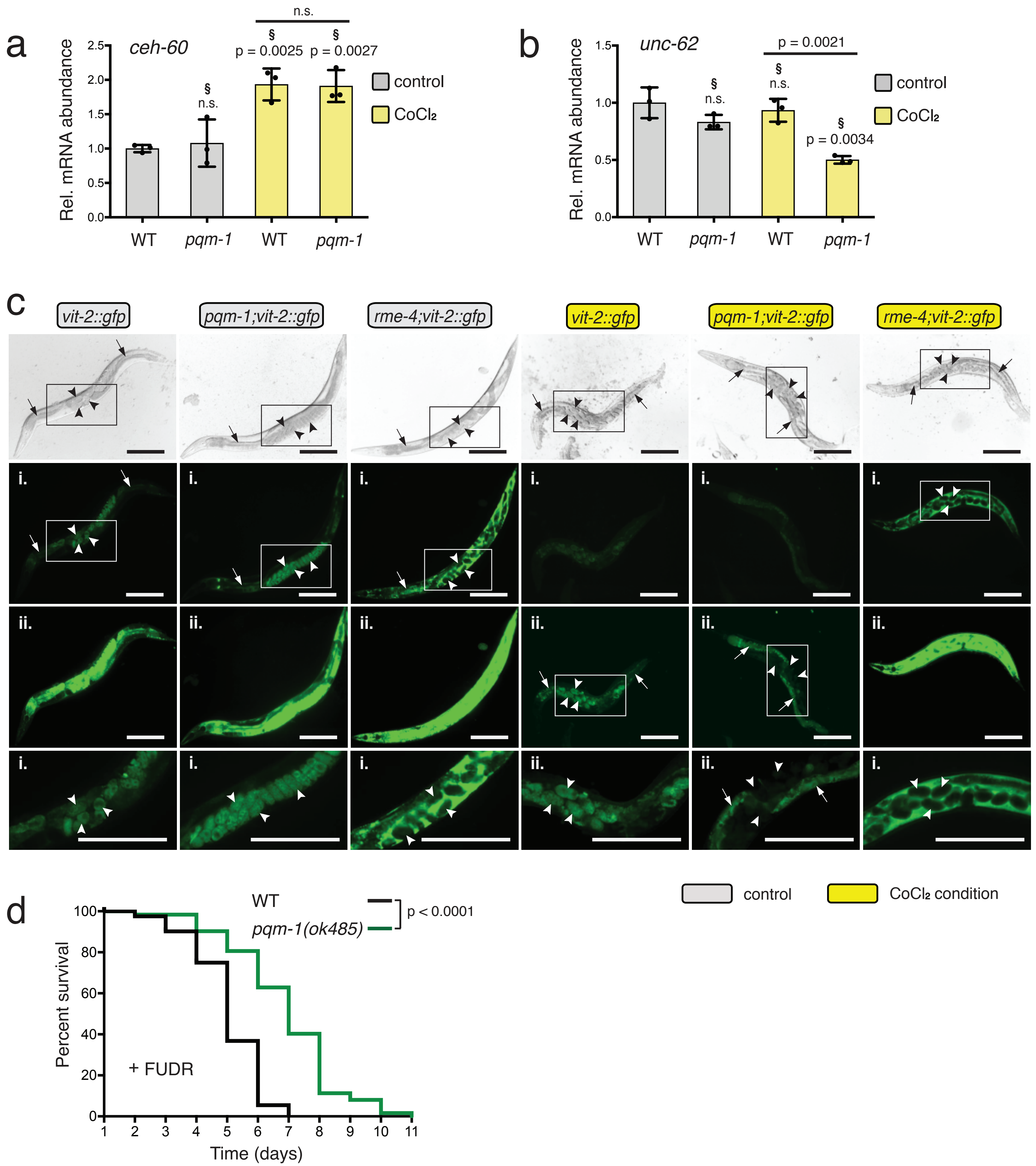

a, b) $CoCl_2$- and *pqm-1*-dependent regulation of endogenous *ceh-60* (a) and *unc-62* (b) determined by RT-qPCR (n = 3 biological replicates) following 5 mM $CoCl_2$ exposure for 20 hr at early adulthood. Two-tailed *t*-test (a, b), mean +/- SD, (§) indicates statistical significance for displayed

conditions versus wild-type control without $CoCl_2$. ns = not significant. c) Effect of *pqm-1* loss on VIT-2::GFP levels in embryos of control worms and worms exposed to 5 mM $CoCl_2$ for 24 hr. i and ii indicate short and long exposure times, respectively. Animals were synchronized at the L4 larval stage for adult analyses. Arrows point to intestine; arrowheads point to embryos; scale bars: 200 μm. d) Survival analysis of animals exposed to 5 mM $CoCl_2$ and 400 μM FUDR at the L4 larval stage. Log-rank analysis was performed, WT (N2) (n = 75), *pqm-1(ok485)* (n = 62), three independent repeats. Source data are provided as a Source Data file for Supplementary Fig. 6a, b, d.

Supplementary Table 1. qRT-PCR primers used in this study

mRNA target	Direction	Primer Sequence (5' to 3')	Source
fat-7	Forward	ATCAACAGCGCTGCTCACTAT	This study
	Reverse	AACGGCGGAAACAGAAGTATC	
sodh-1	Forward	CTGGATGGCAACTTGGAGAC	
	Reverse	AATTCGCAGTTGAGGCAGTT	
unc-62	Forward	AGCGGCGACTCAAAAGTGTG	
	Reverse	CGCTTCTCTCGTTTGATGCTC	
cdc-42	Forward	CTGCTGGACAGGAAGATTACG	Hoogewijs, D. et al. 2008
	Reverse	CTCGGACATTCTCGAATGAAG	
pmp-3	Forward	GTTCCCGTGTTCATCACTCAT	
	Reverse	ACACCGTCGAGAAGCTGTAGA	
Y45F10D.4	Forward	GTCGCTTCAAATCAGTTCAGC	
	Reverse	GTTCTTGTCAAGTGATCCGACA	
vit-1	Forward	GAGGTTCGCTTTGACGGATA	Dowen, R.H., 2019
	Reverse	GGCTTCACATTCCTCGTTCT	
vit-2	Forward	GACACCGAGCTCATCCGCCCA	
	Reverse	TTCCTTCTCTCCATTGACCT	
vit-3,4,5	Forward	CATGTGCACCATCGAAGAACTC	
	Reverse	CCAATGTGGTTTCAATGACAAGTTG	
vit-6	Forward	TTCACCCAGAAGCCAGTTC	
	Reverse	AGGATGGGAGGCAGTAGAC	
ceh-60	Forward	AGTTCTACGGTTGCATCTTCG	
	Reverse	AGTGTGGCTGATGGAGAAAC	
