# Supplementary material for: PQM-1 controls hypoxic survival via regulation of lipid metabolism
Source: Nat Commun. 2020 Oct 2;11:4627. doi: 10.1038/s41467-020-18369-w (PMC7532158; doi:10.1038/s41467-020-18369-w)
Supplement: Supplementary file 2 — Description of Additional Supplementary Information [file 41467_2020_18369_MOESM2_ESM.pdf]

Description of Additional Supplementary Files

File Name: Supplementary Data 1
Description: Significant gene expression changes in wild-type animals exposed to CoCl2 for 6 hr. Gene list was generated by one-class SAM (WT +CoCl2 versus WT -CoCl2). SAM uses twosided statistical testing and reports q-values, which are false discovery rates (multiple comparisons are performed).

File Name: Supplementary Data 2
Description: Significant gene expression changes in pqm-1(ok485) mutants exposed to CoCl2 for 6 hr. Gene list was generated by one-class SAM (pqm-1(ok485) +CoCl2 versus WT +CoCl2). SAM uses two-sided statistical testing and reports q-values, which are false discovery rates (multiple comparisons are performed).

File Name: Supplementary Data 3.
Description: Significant gene expression changes in pqm-1(ok485) mutants versus WT animals exposed to CoCl2 for 6 hr. Gene list was generated by two-class SAM (pqm-1(ok485) +CoCl2 versus WT +CoCl2 compared to pqm-1(ok485) -CoCl2 versus WT -CoCl2). SAM uses twosided statistical testing and reports q-values, which are false discovery rates (multiple comparisons are performed).

File Name: Supplementary Data 4
Description: Significant gene expression changes in pqm-1(ok485) mutants versus WT animals exposed to CoCl2 for 20 hr. Gene list was generated by two-class SAM (pqm-1(ok485) +CoCl2 versus pqm-1(ok485) -CoCl2 compared to WT +CoCl2 versus WT -CoCl2). SAM uses twosided statistical testing and reports q-values, which are false discovery rates (multiple comparisons are performed).
